# Night work, sleep disruption and long-COVID risk: a population-based cohort study

**DOI:** 10.5271/sjweh.4318

**Published:** 2026-11-01

**Authors:** Kyriaki Papantoniou, Ana Espinosa, Marianna Karachaliou, Camille Lassale, Gemma Castaño-Vinyals, Barbara N Harding, Sara De Matteis, Kurt Straif, Eva Alonso-Nogués, Carlota Dobaño, Gemma Moncunill, Natalia Blay, Susana Iraola-Guzmán, Rafael de Cid, Judith Garcia-Aymerich, Manolis Kogevinas

**Affiliations:** 1 Department of Epidemiology, Center for Public Health, Medical University of Vienna, Austria.; 2 Barcelona Institute for Global Health (ISGlobal), Barcelona, Spain.; 3 CIBER Epidemiología y Salud Pública (CIBERESP), Madrid, Spain.; 4 Universitat Pompeu Fabra, Barcelona, Spain.; 5 Erasmus MC, Rotterdam, Netherlands.; 6 CIBER Physiopathology of Obesity and Nutrition (CIBEROBN), Madrid, Spain.; 7 College of Population Health, University of New Mexico, Albuquerque, NM, USA.; 8 Department of Public Health Sciences and Pediatrics, University of Turin, Italy.; 9 Boston College, Chestnut Hill, Massachusetts, USA.; 10 Blood Division, Banc de Sang i Teixits (Bsupplementary table S), Barcelona, Spain.; 11 CIBER Enfermedades Infecciosas (CIBERINFEC), Barcelona, Spain.; 12 Genomes for Life-GCAT Lab, CORE Program. Germans Trias I Pujol Research Institute (IGTP), Badalona, Spain.; 13 Grup de Recerca en Impacte de Les Malalties Cròniques I Les Seves Trajectòries (GRIMTra) (IGTP), Badalona, Spain.; 14 Hospital del Mar Medical Research Institute, Barcelona, Spain.

**Keywords:** insomnia, shift work, SARS-CoV-2, night shift work, sleep disturbance, COVID-19

## Abstract

**Objective:**

Circadian and sleep disruption may increase the risk and severity of SARS-CoV-2 infections, but the role of night shift work and disturbed sleep in the development of long-COVID remains inadequately investigated. We prospectively examined the association of night work and sleep disturbances with risk of long-COVID.

**Methods:**

We followed participants of the COVICAT study, a population-based cohort of adults in Catalonia, Spain between 2021 and 2023. Night shift work and sleep characteristics were assessed in 2021. Long-COVID was defined as the report of persistent symptoms (>3 months) after a SARS-CoV-2 infection. Poisson regression analysis was performed to evaluate the independent and joint effects of night shift work and sleep disturbances including chronic insomnia with risk of long-COVID. Analyses were stratified by sex, body mass index and chronotype.

**Results:**

During the two-year follow-up, 284 of the 1899 newly infected participants reported long-COVID symptoms. Compared to day workers, night shift workers had 88% higher risk of developing long-COVID [95% confidence interval (CI) 1.29–2.72], after adjusting for potential confounders. The risk further increased among night workers with obesity. Chronic insomnia in the general population was associated with 42% increased risk of long-COVID (95% CI 1.12–1.78). Night workers with chronic insomnia had a 2.7 fold higher long-COVID risk (95% CI 1.60–4.51) compared to day workers without chronic insomnia. Associations persisted in various sensitivity analyses.

**Conclusions:**

Our findings from a large prospective cohort indicate that night shift work and poor sleep are independently and jointly associated with a higher risk of long-COVID.

Circadian rhythms and sleep are involved in the regulation of the innate and adaptive immunity, including various functions such as immune cell production and activity (1). Experimental human studies have demonstrated that circadian misalignment and sleep deprivation are associated with impaired immune responses, weaker vaccine immunogenicity and increased susceptibility to infections (2, 3).

Shift workers frequently experience circadian and sleep disruption due to their irregular work schedules and are at an increased risk of respiratory infections (4, 5). In previous observational studies, night shift work has been associated with increased incidence of SARS-CoV-2 infection (6, 7), coronavirus disease 2019 (COVID-19) (8) and COVID-19 related hospitalization (9). Furthermore, workers in occupations involving night work, such as health care and transportation, were found to be at higher long-COVID risk (10). The association between night work and SARS-CoV-2 infection outcomes is biologically plausible. Exposure to pathogens during time windows of higher susceptibility, such as the biological night, leads to worse infection outcomes in experimental animal studies (11). Night workers and sleep deprived individuals may exhibit weaker vaccine induced responses which can lead to lower vaccine efficacy and increased risk of acquiring breakthrough infections, potentially also leading to more severe or persistent infection outcomes (12). To our knowledge no previous study has evaluated the association of night shift work with persistence of symptoms following SARS-CoV-2 acute infection, also known as long-COVID.

Sleep disturbances, such as sleep deprivation or poor sleep quality, are common consequences of night shift work but are also prevalent in the general population. Such disturbances have been associated with increased vulnerability to infections including COVID-19 (13). Short sleep duration emerged as a risk factor for the development and persistence of long-COVID in a recent analysis (14), but sleep quality and sleep timing were not analyzed. A report from a prospective cohort of US registered adult female nurses (mean age 65 years) showed that healthy sleep both before and during the COVID-19 pandemic was protective against long-COVID (15). Replication of these results in other populations including men and younger individuals working in a variety of occupations, as well as evaluation of co-exposure to sleep disturbances and night shift work is warranted to better identify workers with increased susceptibility to infections and post-acute infection sequelae.

We aimed to prospectively evaluate whether exposure to night shift work and sleep disturbances are independently and jointly associated with an increased risk of long-COVID using data from the COVICAT cohort, a population-based cohort of adults in Catalonia, Spain. We hypothesized that night workers and individuals with disturbed sleep would be at higher risk of long-COVID compared to day workers and participants with healthy sleep. We also examined if night work was associated with risk of SARS-CoV-2 infection, as a secondary outcome.

## Methods

This study is based on the COVICAT study, a network of population-based adult cohorts in Catalonia, Spain, established to evaluate the health impacts of the COVID-19 pandemic. We used data from the largest of these, the GCAT|Genomes for Life cohort, a population-based study that recruited 40–65-year-old residents of Catalonia between 2014 and 2017, with >15 years of pre-pandemic follow-up and linkage with electronic health records (EHR) (16) (17).

COVICAT/GCAT comprises three longitudinal data collection waves. The baseline survey (June–November 2020) comprised 10 087 participants and collected data on SARS-CoV-2 testing, symptoms, exposures, and mental and general health; 4100 participants also provided blood samples for serological testing using validated Luminex-based multiplex immunoassays. The first follow-up (June–August 2021; N=8215) updated vaccination status, incident infections, and persistent symptoms, while also collecting detailed demographic, occupational, lifestyle, and health information. The second follow-up (February–May 2023; N=5215; 74% response) focused on reinfections, vaccination status, and long-COVID. At all waves, participants completed a web-based or telephone survey that included questions on socio-demographics, health, behavioral and environmental risk factors. Data on the number of doses, date of administration and trade names of COVID-19 vaccines were retrieved from EHR of the Epidemiological Surveillance Emergency Service of Catalonia of the Department of Health. By late spring 2021, the majority of the population (97%) had received at least one vaccine dose. The study received approval from the local ethics committees at the Hospital Universitari Germans Trias i Pujol (CEI no. PI-20-182) and the Parc de Salut Mar (CEIM-PS MAR, no. 2020/9307/I and CEIMS-PS MAR, no 2020/9160). All participants provided informed consent before participating in the study.

### Exposure assessment

In the 2021 questionnaire (baseline for the present analysis), night shift work and sleep habits were assessed. The questions queried current or recent exposures and changes occurring since the last survey/beginning of the pandemic in March 2020.

Night shift work (defined as ≥3 hours of work during 24:00–06:00 hours) exposure since the beginning of the pandemic was self-reported in 2021 (yes/no). Information on night shift work frequency using a priori defined cut-offs (≥3 versus <3 nights/month) and (recent) duration of night shift work since March 2020 (≥3 versus <3 months) was additionally collected. Pre-pandemic (2018) night shift work status was also assessed to examine past night work history.

Participants reported their current sleep duration (<6, 6, 7, 8, ≥9 hours), sleep problems (difficulty falling asleep, interruptions during sleep, waking up earlier than planned in the morning), occurrence and frequency of sleep problems lasting for ≥3 months (no, <3 versus ≥3 nights/week), sleep medication use (no, natural, medical) and sleep and wake up times on work days and free days. Chronic insomnia was defined as a report of persistent sleep problems (≥3 months) occurring more than 3 nights/week (no, yes). Chronotype was assessed using the Munich Chronotype Questionnaire and calculated as the mid-sleep time on free days corrected for oversleep on free days compared to work days (18).

### Outcome assessment

We defined SARS-CoV-2 infection as: (i) self-reported positive COVID-19 test (polymerase chain reaction or antigen test) *or* self-reported hospital admission due to COVID-19 in the questionnaire *or* ≥4 symptoms *and* contact with infected; (ii) EHR positive SARS-CoV-2 test *or* COVID-19 diagnosis (ICD-10 codes U071, U072, U10 or J1282) in the period between 2021-2023; or (iii) seronegative in 2021 and N-antigen seropositivity in 2023 (14). We classified the severity of SARS-CoV-2 infection according to WHO, based on self-reported information, as asymptomatic (no symptoms reported), mild/moderate (≥1 symptom), severe (hospitalization), and critical (non-invasive or invasive mechanic ventilation, inserted tracheostomy, extracorporeal membrane oxygenation, mechanical ventilation) (19). Using the date of infection, we classified our population as infected with pre-omicron or omicron variants. We considered omicron infections all those occurring after 29 November 2021, as this corresponds to the date of the first omicron case in Catalonia according to the SARSCoV-2 submitted sequences to GISAID (www.gisaid.org; up to 20 April 2023).

Among participants with a new SARS-CoV-2 infection, long-COVID was defined as continuous, relapsing and remitting, or progressive disease state according to the NASEM (20) definition and was ascertained as (i) a self-report of at least one of the following symptoms or sequelae such as fever, loss of appetite, dry cough, dyspnoea/shortness of breath, persistent chest pain/tightness, throat pain and tiredness/unusual fatigue (full list of symptoms are described in the supplementary materials, www.sjweh.fi/article/4318), for a duration of ≥3 months (not necessarily constant and uninterrupted during the 3-month period), which could not be explained by an alternative diagnosis based on responses in the 2023 questionnaire or (ii) identification of a COVID-19 sequelae (ICD-10 B94.8) or long-COVID diagnosis (U09.9) from EHR during the study period (2021–2023).

### Additional data assessment

We obtained information from the 2021 questionnaire on the following individual characteristics: socio-demographics (sex, age, education, housing, region of living); personal habits (tobacco smoking status and intensity, alcohol intake status, amount of physical activity by International Physical Activity Questionnaire category); body mass index (BMI) status was calculated based on self-reported weight (kg) and height (m) as weight/height^2^ and classified as healthy weight (<25 kg/m^2^), overweight (25–29.9 kg/m^2^) and obesity (≥30 kg/m^2^); health status (self-appraisal of health status), presence of any chronic disease that required treatment in the last six months (chronic bronchitis/chronic obstructive pulmonary diseases/emphysema, other respiratory diseases, hypertension, heart disease, diabetes, digestive diseases, chronic liver diseases, gynecological diseases, joint problems/rheumatism, rheumatoid arthritis, other autoimmune diseases, human immunodeficiency virus/other immunodeficiency problems, cancer, depression, anxiety, other mental health problems/addictions, chronic kidney disease) vaccination history (vaccination status, vaccination type, number of vaccine doses) and working in jobs at high COVID-19 risk (based on job titles, job-list based risk and ad hoc job-exposure matrix scores), described in detail elsewhere (10). Job titles were classified according to the International Standard Classification of Occupations 2008 (ISCO-08) system. We additionally assessed psychosocial factors including anxiety and depression using the Hospital Anxiety and Depression Scale (HADS) (21), perceived stress using the Perceived Stress Scale (PSS) (22) and multidimensional perceived social support using the Multidimensional Scale of Perceived Social Support (MSPSS) (23)

### Selection of analytic samples

Figure 1 presents the flow chart for the selection of the analytic samples. This analysis includes participants who completed both the 2021 and 2023 GCAT questionnaires. For the SARS-CoV-2 infection incidence analysis, we included 2941 participants who were not infected before 2021 and had complete data on exposures (night work and sleep characteristics) and key confounders. For the long-COVID analysis, we further restricted the sample to participants who acquired a new SARS-CoV-2 infection between 2021 and 2023, resulting in 1899 individuals. We excluded participants without 2023 questionnaire data (N=4096), those infected before 2021 (N=969), and those with missing exposure (N=155) or confounder information (N=54). Additionally, 1042 participants who did not become infected between 2021 and 2023 were excluded from the long-COVID analysis.

**Figure 1:**
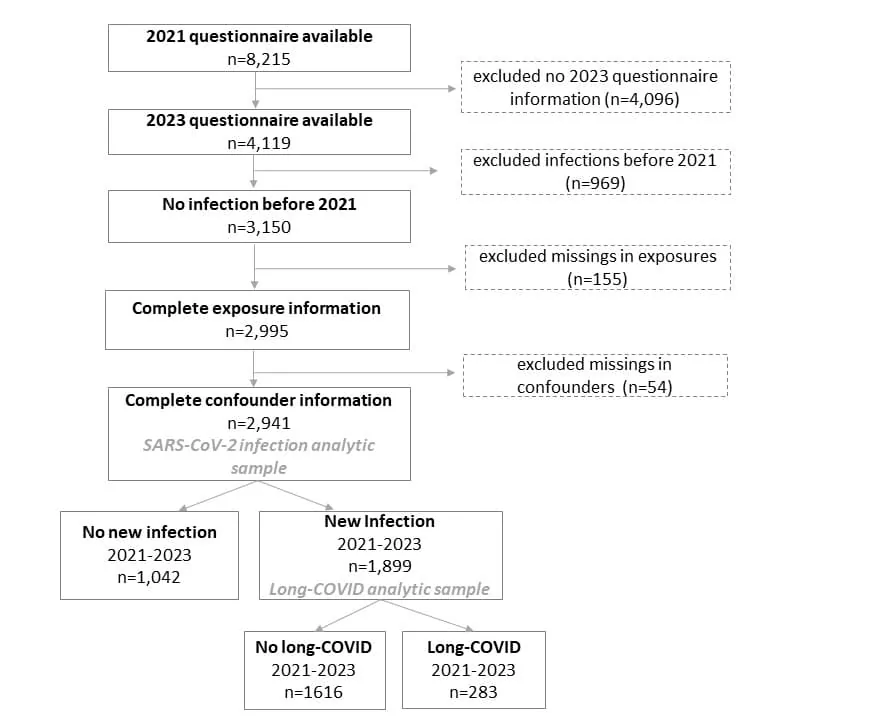
Flow chart of the study population and selection of analytic samples in the COVICAT cohort (2021–2023).

### Statistical analyses

We used Poisson regression models to estimate risk ratios (RR) and 95% confidence intervals (CI) of night work (current night work, night work frequency, night work recent duration, night work past history) and sleep characteristics (current sleep duration, sleep problems and chronotype) in relation to risk of SARS-CoV-2 infection and long-COVID adjusting for potential confounders. Model 1 included age, sex and education; model 2 additionally adjusted for socioeconomic status, ethnicity, smoking, physical activity and BMI and was considered the main model. We additionally adjusted for vaccination history (vaccination type, number of vaccine doses), chronic diseases in the last 6 months, SARS-CoV-2 infection severity, and high risk of COVID-19 job. These variables were considered separately because they may lie in the causal pathway and could act as mediators. Potential confounders were selected a priori based on known and suspected risk factors for SARS-CoV-2 infection and long-COVID including previously described risk and protective factors in the COVICAT cohort (14). Analyses were stratified by sex, obesity (BMI<30, ≥30 kg/m^2^) status and chronotype (early, intermediate, late). Statistical interaction was evaluated using Wald tests of interaction terms included in the models (model 2). Joint effect analyses of night work and sleep characteristics were performed. Departure from additivity was assessed using the relative excess risk due to interaction (RERI) (24). In sensitivity analyses (i) night shift workers were excluded from the sleep and long-COVID analyses, (ii) analyses were restricted to participants who were employed in 2021, (iii) analyses of night work and sleep disturbances with long-COVID were restricted to mild/moderate severity infections and (iv) a stricter definition of long-COVID (≥2 symptoms reported) was used for all the analyses. Last, basic sociodemographic and lifestyle characteristics were compared between those included in our analytic sample and excluded individuals. Analyses were performed using Stata (version 16.1; Stata Corp LP; College Station, Texas, USA). Statistical significance was set at P<0.05.

## Results

### Descriptive analysis

During the 2-year follow-up period, among 2941 participants at risk for SARS-CoV-2 infection, 1899 were newly infected with SARS-CoV-2, of whom 283 developed long-COVID (280 cases ascertained through questionnaire, 3 cases identified through EHR). The vast majority of the infections during this period were mild or moderate (N=1562; 82%), 330 (17.3%) were asymptomatic and only 7 (0.4%) were severe or critical infections. The majority of infections were classified as having occurred during the Omicron wave (93% of infections and long-COVID cases). The three most common symptoms/phenotypes of long-COVID were neurological symptoms and sequelae (54.4%), muscular symptoms and sequelae (30.4%) and respiratory symptoms and sequelae (20.5%).

Night shift workers (N=89 participants, 5.6% of the whole sample), were younger (mean age 53 versus 56 years), less likely to have university education (42% versus 58%), more likely male (62% versus 36%), obese (23% versus 18%) and more likely to report high levels of physical activity (43% versus 33%), compared to day workers (table 1). Night and day workers were employed across a range of occupational sectors, including the healthcare, emergency services and various types of industries (supplementary table S1). The most commonly reported occupations among night workers were police officers (22%), nursing professionals (14%), health care assistants (14%), and mechanical machinery assemblers (11%). Night shift workers were also less likely to be vaccinated with a full vaccination scheme (3–4 doses: 67.4% versus 72.6%), slightly more likely to have a disease that required medical treatment in the last six months (37% versus 34%) but more likely to report excellent health status (9% versus 3%) compared to day workers. Participants with chronic insomnia at baseline (N=365, 19.2% of the whole sample), were more likely to be female (72% versus 61%), less likely to have a university degree (51% versus 58%) and more likely to be current smokers (16% versus 14%), compared to study participants without chronic insomnia (table 1). Participants with short sleep duration (<6 hours) tended to be younger with lower educational level, higher alcohol consumption, and more likely to be overweight or obese, compared to participants with medium sleep duration (6–9 hours) and long (≥9 hours) sleep (supplementary table S2). Late chronotypes tended to be older, and more likely to report overweight and obesity as well as lower levels of physical activity, compared to early chronotypes. Sociodemographic characteristics of the second analytic sample including participants at risk of SARS-CoV-2 infection, are described in supplementary table S3.

**Table 1:** Baseline (2021) characteristics of the study population at risk for long-COVID by night shift work and chronic insomnia categories in the COVICAT cohort (N=1899)*. [IPAQ=International Physical Activity Scale; PSS=Perceived Stress Scale; HADS=Hospital Anxiety and Depression Scale; FSSQ=Functional Social Support Questionnaire]

Characteristic	Night shift work						Chronic insomnia				
	No (N=1810)			Yes (N=89)			No (N=1534)			Yes (N=365)	
	N (%)	Mean (SD)/Median (IQR)		N (%)	Mean (SD)/Median (IQR)		N (%)	Mean (SD)/Median (IQR)		N (%)	Mean (SD)/Median (IQR)
Age (years)		55.9 (6.9)			52.7 (6.3)			55.9 (7.0)			55.2 (6.5)
Gender											
Male	643 (35.5)			55 (61.8)			594 (38.7)			104 (28.5)	
Female	1167 (64.5)			34 (38.2)			940 (61.3)			261 (71.5)	
Educational level											
University	1041 (57.5)			37 (41.6)			891 (58.1)			187 (51.2)	
Secondary	636 (35.1)			45 (50.6)			541 (35.3)			140 (38.4)	
Primary or less	133 (7.3)			7 (7.9)			102 (6.6)			38 (10.4)	
Smoking status											
Never smoker	781 (43.1)			39 (43.8)			683 (44.5)			137 (37.5)	
Ex-smoker	779 (43.0)			35 (39.3)			643 (41.9)			171 (46.8)	
Smoker	250 (13.8)			15 (16.9)			208 (13.6)			57 (15.6)	
Current alcohol consumption											
No	406 (22.4)			21 (23.6)			319 (20.8)			108 (29.6)	
Yes, occasionally	1180 (65.2)			60 (67.4)			1027 (67.0)			213 (58.4)	
Yes, daily	223 (12.3)			8 (9.0)			187 (12.2)			44 (12.1)	
Physical activity (IPAQ scoring)											
Low	351 (19.4)			14 (15.7)			283 (18.4)			82 (22.5)	
Moderate	867 (47.9)			37 (41.6)			736 (48.0)			168 (46.0)	
High	592 (32.7)			38 (42.7)			515 (33.6)			115 (31.5)	
Body mass index (BMI) (kg/m^2^)		26.3 (4.4)			27.0 (4.7)			26.3 (4.4)			26.3 (4.6)
BMI categorical (kg/m^2^)											
<25	791 (43.7)			34 (38.2)			662 (43.2)			163 (44.7)	
25–29.9	698 (38.6)			35 (39.3)			600 (39.1)			133 (36.4)	
≥30	321 (17.7)			20 (22.5)			272 (17.7)			69 (18.9)	
COVID-19 severity among infected											
Asymptomatic	310 (17.1)			20 (22.5)			272 (17.7)			58 (15.9)	
Mild/moderate	1494 (82.5)			68 (76.4)			1257 (81.9)			305 (83.6)	
Severe/critical	6 (0.3)			1 (1.1)			5 (0.3)			2 (0.5)	
Vaccination doses (pre-infection)											
No vaccine	103 (5.8)			5 (5.6)			85 (5.6)			23 (6.4)	
1 dose	18 (1.0)			3 (3.4)			18 (1.2)			3 (0.8)	
2 doses	367 (20.6)			21 (23.6)			306 (20.2)			82 (22.8)	
3–4 doses	1294 (72.6)			60 (67.4)			1103 (72.9)			251 (69.9)	
Type of first vaccine											
No vaccine	103 (5.7)			5 (5.6)			85 (5.5)			23 (6.3)	
BioNTech	858 (47.4)			44 (49.4)			728 (47.5)			174 (47.7)	
Moderna	205 (11.3)			17 (19.1)			167 (10.9)			55 (15.1)	
Oxford	612 (33.8)			22 (24.7)			533 (34.7)			101 (27.7)	
J&J / Sinovac	32 (1.8)			1 (1.1)			21 (1.4)			12 (3.3)	
Health state											
Excellent	82 (4.5)			8 (9.0)			85 (5.5)			5 (1.4)	
Very good	579 (32.0)			27 (30.3)			533 (34.7)			73 (20.0)	
Good	902 (49.8)			37 (41.6)			752 (49.0)			187 (51.2)	
Fair	225 (12.4)			17 (19.1)			156 (10.2)			86 (23.6)	
Poor	22 (1.2)			0 (0.0)			8 (0.5)			14 (3.8)	
Disease that required medical treatment for >6 months	605 (33.9)			32 (37.2)			479 (31.7)			158 (43.9)	
PSS10: total score		13.5 (7.2)			13.4 (7.3)			12.4 (6.7)			18.1 (7.8)
HADS: Anxiety subscale		5 (5)			5 (5)			5 (4)			8 (7)
HADS: Depression subscale		3.7 (3.7)			4.1 (3.9)			2 (4)			5 (7)
FSSQ: Social Support Score		26.6 (5.8)			24.4 (6.1)			29 (8)			26 (10)

* Numbers may not add up to the total due to missing data.

### Night shift work and risk of SARS-CoV-2 and long COVID

Night shift work at baseline was not associated with a higher risk of SARS-CoV-2 infection during the follow-up period (2021–2023) (supplementary table S4). Among participants with a SARS-CoV-2 infection, working night shifts at baseline was associated with higher risk of developing long-COVID (RR 1.88; 95% CI 1.29–2.72) compared to day work, after adjusting for potential confounders (table 2). Additional adjustments for vaccination history, chronic diseases and disease severity did not materially change the results (supplementary table S5). Adjustments for psychosocial factors attenuated the association of night work with long-COVID but effect estimates remained statistically significant (supplementary table S6). Further adjustment for high risk for COVID-19 jobs in a subset with this information, slightly attenuated the magnitude of the effect of night work on long-COVID (supplementary table S7). When considering frequency and duration of night short work, a higher risk of long-COVID was observed among participants with more intense night shift schedules (>3 nights/month RR 2.07; 95% CI 1.41–3.03) and longer duration of recent exposure (≥3 months: RR 2.07; 95% CI 1.41–3.03) (table 2). Both current exposure (night work in 2021) and past exposure history (current and pre-pandemic night work) were associated with long-COVID risk among participants with information at both time points. In stratified analyses effect estimates did not differ between sexes, but the risk tended to be higher among individuals with obesity (RR 3.77; 95% CI 2.40–5.91) compared to those with a BMI of <30 kg/m^2^ (RR 1.28; 95% CI 0.72–2.27, P-value for interaction=0.003) (table 3). The association between night shift work and long-COVID was stronger in both morning chronotypes (RR 2.69; 95% CI 1.40–5.15) and in evening chronotypes (RR 1.92; 95% CI 1.16–3.17), compared to participants with intermediate chronotypes (RR 1.11; 95% CI 0.44–2.76) (P-value for interaction=0.29) (supplementary table S8).

**Table 2:** Association of night shift work, sleep disturbances and long–COVID among SARS–CoV–2 infected participants in the COVICAT cohort (2021–2023) (N=1899)

	No long-COVID (N=1616)		Long-COVID (N=283)	RR (95% CI) ^1^	RR (95% CI) ^2^
	N (%)		N (%)		
**Night shift work**					
Night shift work status (2021)					
No	1549 (95.9)		261 (92.2)	ref.	ref.
Yes	67 (4.1)		22 (7.8)	1.89 (1.28–2.77)	1.88 (1.29–2.72)
Night shift work frequency					
No	1549 (95.9)		261 (92.2)	ref.	ref.
<3 nights/month	16 (1.0)		3 (1.1)	1.21 (0.43–3.40)	1.19 (0.41–3.42)
≥3 nights/month	51 (3.2)		19 (6.7)	2.07 (1.38–3.10)	2.07 (1.41–3.03)
Night shift duration since 2020					
No	1549 (95.9)		261 (92.2)	ref.	ref.
<3 months	22 (1.4)		5 (1.8)	1.51 (0.67–3.39)	1.43 (0.63–3.23)
≥3 months	45 (2.8)		17 (6.0)	2.03 (1.34–3.09)	2.06 (1.39–3.06)
Night shift work history ^3^					
No	1549 (97.1)		261 (93.9)	ref.	ref.
Yes, current, no pre–pandemic	19 (1.2)		7 (2.5)	1.97 (1.05–3.68)	1.96 (1.07–3.58)
Yes, current and pre–pandemic	28 (1.8)		10 (3.6)	1.93 (1.11–3.33)	1.93 (1.15–3.23)
**Sleep**					
Sleep duration (hours)					
<6	138 (8.5)		36 (12.7)	1.42 (1.01–1.99)	1.35 (0.97–1.89)
6–7	477 (29.5)		86 (30.4)	1.09 (0.84–1.41)	1.04 (0.80–1.35)
7–8	676 (41.8)		107 (37.8)	ref.	ref.
8–9	294 (18.2)		44 (15.5)	0.94 (0.67–1.30)	0.92 (0.67–1.28)
≥9	31 (1.9)		10 (3.5)	1.80 (1.00–3.24)	1.61 (0.88–2.93)
Sleep problems frequency					
No	1025 (62.3)		139 (47.4)	ref.	ref.
Yes, <3 nights/week	324 (19.7)		73 (24.9)	1.45 (1.12–1.88)	1.44 (1.11–1.87)
Yes, ≥3nights/week	297 (18.0)		81 (27.6)	1.64 (1.27–2.11)	1.59 (1.24–2.04)
Chronic insomnia					
No	1349 (82.0)		212 (72.4)	ref.	ref.
Yes	297 (18.0)		81 (27.6)	1.46 (1.15–1.84)	1.42 (1.12–1.78)
Sleep medication					
No	1332 (82.4)		209 (73.9)	ref.	ref.
Yes, natural treatment	146 (9.0)		43 (15.2)	1.55 (1.15–2.10)	1.55 (1.15–2.09)
Yes, medical treatment	138 (8.5)		31 (11.0)	1.30 (0.93–1.83)	1.22 (0.86–1.73)
Sleep timing/chronotype (MSF) ^4^					
Tertile 1 (early)	645 (39.9)		93 (32.9)	ref.	ref.
Tertile 2 (intermediate)	446 (27.6)		82 (29.0)	1.24 (0.95–1.63)	1.25 (0.96–1.65)
Tertile 3 (late)	525 (32.5)		108 (38.2)	1.36 (1.05–1.75)	1.32 (1.02–1.70)

^1^ Model 1 adjusted for age (continuous), sex and education (university/secondary/primary or less). ^2^ Model 1 + adjustment for smoking (never/ex/current smoker), physical activity according to IPAQ rules (low/moderate/high) and body mass index (continuous). ^3^ Among workers with complete shift work information at both time points. ^4^ Mid–sleep on free days (MSF) is calculated based on self–reported time of going to bed/waking up on a free day corrected for overlseep.

**Table 3:** Multivariable adjusted association of night work and sleep with long-COVID, by sex and body mass index (BMI) categories, in the COVICAT cohort (2021–2023) (N=1899). [RR=risk ratio; CI=confidence interval.]

	Men (N=698)		Women (N=1201)	Interaction P–value ^5^	BMI<30 kg/m^2^ (N=1558)		BMI≥30 kg/m^2^ (N=341)	Interaction P–value ^5^
	RR (95% CI) ^1^		RR (95% CI) ^1^		RR (95% CI) ^2^		RR (95% CI) ^2^	
**Night shift work**								
Night shift work status (2021)								
No	Reference		Reference	0.886	Reference		Reference	0.003
Yes	1.93 (1.09–3.41)		1.83 (1.12–2.98)		1.28 (0.72–2.27)		3.77 (2.40–5.91)	
**Sleep**								
Sleep duration (hours) ^3^								
<6	2.20 (1.19–4.07)		1.12 (0.75–1.68)	0.060	1.43 (0.96–2.11)		1.18 (0.62–2.28)	0.508
6–7	1.07 (0.61–1.90)		1.03 (0.77–1.38)		1.02 (0.75–1.39)		1.05 (0.64–1.71)	
7–8	Reference		Reference		Reference		Reference	
8–9	1.13 (0.56–2.28)		0.87 (0.60–1.25)		0.99 (0.68–1.42)		0.73 (0.36–1.47)	
≥9	4.39 (1.87–10.33)		0.98 (0.43–2.20)		2.30 (1.25–4.25)		0.49 (0.08–2.92)	
Sleep problems frequency								
No	Reference		Reference	0.002	Reference		Reference	0.056
Yes, <3 nights/week	2.17 (1.23–3.81)		1.32 (0.99–1.77)		1.37 (1.00–1.87)		1.91 (1.20–3.03)	
Yes, ≥3 nights/week	3.44 (2.12–5.58)		1.25 (0.93–1.67)		1.78 (1.34–2.37)		1.08 (0.61–1.92)	
Chronic insomnia								
No	Reference		Reference	0.001	Reference		Reference	0.049
Yes	2.79 (1.80–4.33)		1.14 (0.86–1.50)		1.61 (1.24–2.09)		0.88 (0.51–1.51)	
Sleep medication								
No	Reference		Reference	0.012	Reference		Reference	0.715
Yes, natural treatment	3.33 (1.89–5.85)		1.25 (0.89–1.76)		1.60 (1.13–2.25)		1.46 (0.81–2.64)	
Yes– medical treatment	1.74 (0.87–3.47)		1.09 (0.73–1.61)		1.35 (0.92–1.98)		0.97 (0.48–1.97)	
Sleep timing/chronotype (MSF) ^4^								
Tertile 1 (early)	Reference		Reference	0.158	Reference		Reference	0.182
Tertile 2 (intermediate)	0.78 (0.43–1.40)		1.47 (1.08–2.02)		1.11 (0.82–1.52)		1.91 (1.06–3.42)	
Tertile 3 (late)	1.03 (0.63–1.70)		1.46 (1.08–1.96)		1.18 (0.88–1.58)		2.03 (1.18–3.50)	

^1^ Adjusted for age (continuous), education (university/secondary/primary or less), smoking (never/ex/current smoker), physical activity according to IPAQ rules (low/moderate/high) and body mass index (continuous). ^2^ Adjusted for age (continuous), sex, education (university/secondary/primary or less), smoking (never/ex/current smoker) and physical activity according to IPAQ rules (low/moderate/high). ^3^ Self–reported number of hours of sleep. ^4^ Mid–sleep on free days (MSF) is calculated based on self–reported time of going to bed/waking up on a free day corrected for oversleep. ^5^ P–for interaction obtained from Wald test of the interaction term.

### Sleep health and risk of SARS-CoV-2 and long COVID

No sleep characteristic was associated with risk of SARS-CoV-2 infection, except later chronotypes showed a higher risk of SARS-CoV-2 infection compared to earlier chronotypes (supplementary table S4). Chronic insomnia at baseline (sleep problems for >3 months ≥3 nights/week) was a risk factor for long-COVID (RR 1.42; 95% CI 1.12–1.78) (table 2). Participants who used sleep medication had a lower risk for long-COVID, compared to those who used a natural treatment for their sleep problem. We observed a U-shape association between sleep duration and risk of long-COVID but differences between groups did not reach statistical significance. Later sleep timing/chronotype (top tertile) was associated with an increased risk for long-COVID (RR 1.32; 95% CI 1.02–1.70), compared to early chronotype (bottom tertile). Associations of sleep disturbances with long-COVID risk were more pronounced among men and participants with a normal weight (table 3). Additional adjustment for social support and particularly psychosocial factors such as perceived stress, anxiety and depression scales strongly attenuated these association analyses and, in some cases, statistical significance was lost (supplementary table S6).

### Additional analyses

Joint effects analysis revealed that co-exposure to night work and chronic insomnia was associated with a 2.7 fold increase in risk of long-COVID (RR 2.69; 95% CI 1.60–4.51), compared to participants without night shift work or chronic insomnia, indicating an additive effect (table 4).

**Table 4:** Association of night work with long-COVID in the COVICAT cohort (2021–2023) stratified by chronic insomnia and joint effects of night work and chronic insomnia. [RR=risk ratio; CI=confidence interval.]

	No long-COVID (N=1616)		Long-COVID (N=283)	RR (95% CI)^1^
	N (%)		N (%)	
**Night shift work**				
No chronic insomnia				
No night work	1272 (95.8)		191 (92.7)	
Night work	56 (4.2)		15 (7.3)	1.81 (1.14–2.89)
Chronic insomnia				
No night work	277 (96.2)		70 (90.9)	
Night work	11 (3.8)		7 (9.1)	1.92 (1.11–3.32)^2^
**Joint effects**				
No night work and no chronic insomnia	1272 (78.7)		191 (67.5)	Reference
No night work and chronic insomnia	277 (17.1)		70 (24.7)	1.40 (1.09–1.80)
Night work and no chronic insomnia	56 (3.5)		15 (5.3)	1.81 (1.14–2.89)
Night work and chronic insomnia	11 (0.7)		7 (2.5)	2.69 (1.60–4.51)

^1^ Adjusted for age (continuous), sex, education (university/secondary/primary or less), smoking (never/ex/current smoker), physical activity according to IPAQ rules (low/moderate/high) and body mass index (continuous). ^2^ Interaction P-value=0.877.

In sensitivity analyses, the effects of poor sleep on long-COVID remained consistent with the main analysis after excluding current night shift workers (supplementary table S9). Results were somewhat attenuated but consistent in direction, magnitude and significance when restricting to participants who were employed in 2021 (supplementary table S10). Findings were unchanged after limiting analyses to mild/moderate severity infections (supplementary table S11) or after restricting to participants reporting at least two long-COVID symptoms (supplementary table S12). Characteristics of included and excluded participants were comparable (supplementary table S13).

## Discussion

Night shift work was associated with a higher risk of long-COVID but not with risk of SARS-CoV-2 infection. Associations were stronger among participants with obesity and persisted after various adjustments for confounders. Sleep problems, such as chronic insomnia, were associated with an increased long-COVID risk. Night work and poor sleep synergistically increased the risk of long-COVID. Our findings indicate that night work and poor sleep are independently and jointly associated with long-COVID risk, highlighting a potential role for circadian and sleep disruption in the progression from acute infection to persistent post-infectious sequelae.

We observed that night shift work was a strong risk factor for long-COVID, whereas no association was found with risk or severity of SARS-CoV-2 infection. An elevated risk of long-COVID was observed with higher frequency and duration of night work exposure and among both current and more experienced night shift workers. These findings suggest that the association between night work and long-COVID is unlikely to be explained solely by transient changes in working patterns during the pandemic and may reflect more sustained exposure to night shift work. Although no study has previously evaluated night work in relation to long-COVID, a growing body of literature suggests an increased risk of SARS-CoV-2 infection associated with night work (8), especially in studies conducted during the first year of the pandemic (8). Analyses in the UK Biobank study showed positive associations of night shift work with risk of SARS-CoV-2 infection (6) and COVID-19 hospitalization (9), suggesting that the link between night work and long-COVID could be explained by higher infection susceptibility. However, we did not observe an association between night work and risk of SARS-CoV-2 infection suggesting that circadian disruption may influence the transition from acute infection to chronic post-infectious symptoms rather than simply susceptibility to initial infection. Furthermore, compared to previous studies, ours was conducted in a population with a high vaccination coverage during later stages of the pandemic (2021-2023), characterized by different distribution of circulating variants (omicron period), increased immunity at the population level due to previous infections and vaccination and a predominance of mild or less severe infections (25). In line with our results, a more recent large prospective cohort with multiple exposure measurements during the pandemic showed no association between night-shift work and SARS-CoV-2 infection incidence (7). Furthermore, an analysis of 7141 workers across 15 countries failed to identify an association between shift work and infection risk but demonstrated a higher risk of more severe disease outcomes among night shift workers (26). In our study population, the number of participants with severe SARS-CoV-2 infection was too small to allow for separate subgroup analyses. Taken together, our findings suggest that night work may contribute to a higher risk of long-COVID following mild to moderate SARS-CoV-2 infection, potentially through disruption of recovery processes and facilitation of the progression from acute infection to chronic post-infectious sequelae.

Furthermore, we found that co-exposure to night shift work and persistent sleep problems was associated with even higher risk for long-COVID, although these results were based on small numbers and should be interpreted with caution. This is in line with previous literature showing that joint exposure to night work and poor sleep further increases the risk for respiratory infections (27) as well as chronic disease risk (28–30). Poor sleep quality and acute sleep loss are common consequences of night shift work and sleep disturbances may therefore represent intermediate factors in the causal pathway linking night work to long-COVID. Furthermore, our findings suggest that maladaptation to night shift work, as indicated by the report of clinically significant sleep disturbances such as chronic insomnia, may serve as a marker of increased susceptibility to long-COVID (31). In this context, persistent and chronic sleep problems may not only represent a consequence of night shift work but also indicate underlying individual vulnerability to adverse long-term outcomes following SARS-CoV-2 infection. Last, we observed that the increased long-COVID risk was higher in night shift workers with obesity, even after adjusting for physical activity. Because the subgroup analyses involved a limited number of participants, the observed associations should be interpreted with caution and require replication. Night work and obesity have been independently and jointly associated with a markedly higher COVID-19 risk (32). A recent multi-cohort study showed robust associations between adult obesity and an increased risk of severe infections across diverse pathogen types and populations (33). Obesity can restrict ventilation, impairs immune responses to viral infections, is pro-inflammatory and induces diabetes and cardiovascular dysfunction (34–36). Our results suggest that both obesity and disturbed sleep may contribute to increased susceptibility to long-COVID among night shift workers. This underscores the need for personalized workplace interventions that minimize circadian misalignment and improve sleep with the aim of reducing the risk of metabolic diseases and post-acute infection syndromes.

We also found that sleep problems were independently associated with long-COVID in the general population as indicated by the persistence of results after exclusion of current night workers. We observed increased risk of long-COVID among subjects with pre-existing chronic insomnia symptoms, assessed before the development of long-COVID symptoms. Sleep duration was suggestively associated in a U-shape manner with incident long-COVID, in line with previous results on determinants of health outcomes including persistent long-COVID in the same cohort (14). Adjusting for perceived stress, anxiety and depression, attenuated the associations of sleep disturbances with long-COVID, suggesting that psychosocial factors and stress may contribute to the observed association Because sleep and mental health were assessed at the same time point in our study, both confounding and mediation by poor mental health are plausible explanations for the observed associations. Although poor sleep was not a major risk factor for SARS-CoV-2 infection in our data, emerging evidence shows that unhealthy sleep characteristics are associated with long-COVID risk (37) as well as COVID-19 risk and severity (13, 38, 39). Recently multidimensional sleep health was found to be protective for long-COVID among older female nurses (15). Similarly, retrospectively recalled pre-pandemic insomnia was a risk factor for long-COVID in a survey conducted in 2021, among 15,813 participants across 16 countries (40). In a US nationwide sample, insomnia was associated with prolonged COVID-19 duration suggesting that it may delay individuals’ disease recovery and potentially development of persistent symptoms (41). Given the increase of clinically relevant insomnia prevalence, there is a need to better understand the short- and long-term effects of disturbed sleep on long-COVID and other post-acute infection diseases such as chronic fatigue syndrome (42) (43). These findings also urge the evaluation of the bidirectional association of sleep disturbances and infection related outcomes and underlying pathophysiological mechanisms. From a public health perspective, our results support the importance of promoting sleep hygiene as a low-cost, scalable preventive strategy to reduce risk and potentially mitigate longer-term health consequences following SARS-CoV-2 and other infections. This might be particularly relevant in the context of long-COVID, where preventive strategies remain limited. We therefore emphasize the need for broader sleep health promotion alongside occupational interventions targeting shift work.

Several biological pathways have been proposed whereby circadian and sleep disruption may increase the risk for long-COVID. First, circadian misalignment and sleep deprivation may affect the ability of clearing the infection (eg, through effects on cytokines, monocytes related to phagocytic activity, NK cells and T-cells responses) (1, 2). Second, night workers are more likely to acquire the infection during the night and time of day of encountering a pathogen may modulate host immune responses and infection outcomes (11) (44). Third, night workers may exhibit weaker responses after vaccination which can translate to lower protection levels as is postulated for the meningococcal C (12) and other vaccine types (45–47). Furthermore, sleep deprived individuals experience reduced vaccine-induced humoral immune responses following influenza and hepatitis vaccination (48). Fourth, many components of the immune system and multiple drug targets, exhibit circadian variation under the control of the molecular clock (49). Therefore, night shift workers and individuals with sleep disruption may experience immune deregulation such as immune suppression and impaired vaccine and drug efficacy (50–52). Fifth, night workers experience lower levels of nocturnal melatonin production (53), which has been shown to have anti-inflammatory and antioxidant properties and mitigates mitochondrial dysfunction (54). Last, night work and disturbed sleep are risk factors for many chronic diseases (eg, obesity, type II diabetes, cardiovascular disease) that are predisposing factors for long-COVID (14). These comorbidities, coupled with impaired innate immune defenses (eg, reduced mucociliary clearance and altered viral recognition pathways), may exacerbate the severity of illness or hinder recovery from infection. Our analyses accounted for the report of chronic diseases and results persisted after adjustments suggesting that mediation by comorbidities was unlikely in our data. Collectively, these mechanisms highlight the need to consider sleep and circadian health in strategies to optimize vaccine protection and reduce infection-related risks, particularly in vulnerable occupational groups.

Our study has several strengths. First, the prospective study design that reduces the potential for reverse causation explaining our results. Second, the cases examined in our study were drawn from a well identified population-based cohort with individual level information on exposure, outcomes and key confounders allowing for control for confounding. Third, the comprehensive use of long-COVID symptoms together with the electronic health data integration, and use of international standard criteria to define long-COVID and sensitivity analysis using a stricter definition to assess greater number of symptoms. Fourth, the use of detailed shift work information including pre-pandemic data, and sleep information that allowed for a refined exposure assessment minimizing exposure misclassification. The study also has some limitations. First, our study population consists of working age individuals (40–65 years) which is appropriate for the study of an occupational exposure like night shift work but our results, especially the sleep analyses, may not directly extend to younger or older individuals. Second, our questionnaire did not ascertain information on weekly or monthly working hours and therefore we could not control for overtime work, which often co-occurs or is inherent in occupations with night work and could partly explain the results. Third, although the associations remained robust after adjustment for occupations at high risk of SARS-CoV-2 exposure, we cannot completely exclude the possibility that other occupation-related factors, including work-related stress, may have contributed to the observed risks. Fourth, our outcomes definition and risk estimates relied on contacts through the 2021 and 2023 surveys. This may slightly underestimate the true outcome prevalence if participants developed symptoms between surveys but did not report them. However, any such misclassification would likely be non-differential and would tend to bias our results towards the null. Last, long-COVID symptoms were only asked among infected individuals, which may have led to underestimation of long-COVID cases but increased specificity of the results. While the participation rate declined over time, characteristics of individuals lost to follow-up were not markedly dissimilar to those remaining in the study, minimizing potential biases.

This study is the first to demonstrate an elevated risk of long-COVID among night shift workers, identifying irregular work schedules as a potential determinant of post-acute sequelae of SARS-CoV-2 infection. Concurrent exposure to night work and poor sleep appeared to further increase the risks. The absence of an association with infection risk suggests that circadian disruption and disturbed sleep may play a role in the transition from acute SARS-CoV-2 infection to chronic post-infectious symptoms. Furthermore, sleep disturbances such as chronic insomnia emerged as independent risk factor for long-COVID. These findings underscore the importance of circadian and sleep health in mitigating long-term consequences of COVID-19 among shift workers and the general population. Further research should elucidate causal pathways and delineate the biological and behavioral mechanisms underlying these associations. Personalized prevention strategies aimed at optimizing work schedules, improving sleep quality, and minimizing circadian disruption, particularly among night-shift workers, may help lower the risk of long-COVID.

## Supplementary material

Supplementary material 1 Supplementary material

## References

[1] Haspel JA, Anafi R, Brown MK, Cermakian N, Depner C, Desplats P et al. Perfect timing: circadian rhythms, sleep, and immunity - an NIH workshop summary. JCI Insight 2020 Jan;5(1):e131487. 10.1172/jci.insight.131487 31941836 PMC7030790

[2] Cermakian N, Stegeman SK, Tekade K, Labrecque N. Circadian rhythms in adaptive immunity and vaccination. Semin Immunopathol 2022 Mar;44(2):193–207. 10.1007/s00281-021-00903-7 34825270

[3] Schmitz NC, van der Werf YD, Lammers-van der Holst HM. The Importance of Sleep and Circadian Rhythms for Vaccination Success and Susceptibility to Viral Infections. Clocks Sleep 2022 Feb;4(1):66–79. 10.3390/clockssleep4010008 35225954 PMC8884008

[4] Prather AA, Carroll JE. Associations between sleep duration, shift work, and infectious illness in the United States: Data from the National Health Interview Survey. Sleep Health 2021 Oct;7(5):638–43. 10.1016/j.sleh.2021.05.004 34193397

[5] Loef B, van Baarle D, van der Beek AJ, Sanders EA, Bruijning-Verhagen P, Proper KI. Shift Work and Respiratory Infections in Health-Care Workers. Am J Epidemiol 2019 Mar;188(3):509–17. 10.1093/aje/kwy258 30475977 PMC6395171

[6] Fatima Y, Bucks RS, Mamun AA, Skinner I, Rosenzweig I, Leschziner G et al. Shift work is associated with increased risk of COVID-19: findings from the UK Biobank cohort. J Sleep Res 2021 Oct;30(5):e13326. 10.1111/jsr.13326 33686714 PMC8250353

[7] Loef B, Dollé ME, Proper KI, van Baarle D, Initiative LC, van Kerkhof LW. Night-shift work is associated with increased susceptibility to SARS-CoV-2 infection. Chronobiol Int 2022 Aug;39(8):1100–9. 10.1080/07420528.2022.2069031 35502475

[8] Loef B, Bosma E, van Kerkhof LW, Proper KI, van Baarle D, Dollé ME. Night-shift work and susceptibility to infectious diseases: a systematic review and meta-analysis. Scand J Work Environ Health 2025 Jul;51(4):298–311. 10.5271/sjweh.4225 40188463 PMC12281634

[9] Maidstone R, Anderson SG, Ray DW, Rutter MK, Durrington HJ, Blaikley JF. Shift work is associated with positive COVID-19 status in hospitalised patients. Thorax 2021 Jun;76(6):601–6. 10.1136/thoraxjnl-2020-216651 33903187 PMC8098298

[10] De Matteis S, Consonni D, Espinosa A, de Cid R, Magriña NB, Castaño-Vinyals G et al. Occupational determinants of Long COVID in the population-based COVICAT cohort. Occup Environ Med 2026 Feb;82(12):579–88. 10.1136/oemed-2025-110398 41360602

[11] Sengupta S, Tang SY, Devine JC, Anderson ST, Nayak S, Zhang SL et al. Circadian control of lung inflammation in influenza infection. Nat Commun 2019 Sep;10(1):4107. 10.1038/s41467-019-11400-9 31511530 PMC6739310

[12] Ruiz FS, Rosa DS, Zimberg IZ, Dos Santos Quaresma MV, Nunes JO, Apostolico JS et al. Night shift work and immune response to the meningococcal conjugate vaccine in healthy workers: a proof of concept study. Sleep Med 2020 Nov;75:263–75. 10.1016/j.sleep.2020.05.032 32866895

[13] Liu Z, Luo Y, Su Y, Wei Z, Li R, He L et al. Associations of sleep and circadian phenotypes with COVID-19 susceptibility and hospitalization: an observational cohort study based on the UK Biobank and a two-sample Mendelian randomization study. Sleep 2022 Jun;45(6):zsac003. 10.1093/sleep/zsac003 35034128 PMC8807236

[14] Kogevinas M, Karachaliou M, Espinosa A, Iraola-Guzmán S, Castaño-Vinyals G, Delgado-Ortiz L et al. Risk, determinants, and persistence of long-COVID in a population-based cohort study in Catalonia. BMC Med 2025 Mar;23(1):140. 10.1186/s12916-025-03974-7 40082863 PMC11907888

[15] Wang S, Huang T, Weisskopf MG, Kang JH, Chavarro JE, Roberts AL. Multidimensional Sleep Health Prior to SARS-CoV-2 Infection and Risk of Post-COVID-19 Condition. JAMA Netw Open 2023 May;6(5):e2315885. 10.1001/jamanetworkopen.2023.15885 37252741 PMC10230315

[16] Obón-Santacana M, Vilardell M, Carreras A, Duran X, Velasco J, Galván-Femenía I et al. GCAT|Genomes for life: a prospective cohort study of the genomes of Catalonia. BMJ Open 2018 Mar;8(3):e018324. 10.1136/bmjopen-2017-018324 29593016 PMC5875652

[17] Blay N, Farré X, Garcia-Aymerich J, Castaño-Vinyals G, Kogevinas M, de Cid R. Pre-pandemic disease trajectories and genetic insights into long COVID susceptibility. BMC Med 2025 Oct;23(1):590. 10.1186/s12916-025-04427-x 41146089 PMC12560560

[18] Roenneberg T, Kuehnle T, Juda M, Kantermann T, Allebrandt K, Gordijn M et al. Epidemiology of the human circadian clock. Sleep Med Rev 2007 Dec;11(6):429–38. 10.1016/j.smrv.2007.07.005 17936039

[19] WHO. WHO COVID-19 Clinical management: living guidance. Geneva: WHO; Report No: WHO/2019-nCoV/clinical/20212. 2020 May.

[20] National Academies of Sciences, Engineering, and Medicine; Health and Medicine Division; Board on Global Health; Board on Health Sciences Policy; Committee on Examining the Working Definition for Long COVID. A Long COVID Definition: Chronic A; Systemic Disease State with Profound Consequences. Goldowitz I, Worku T, Brown L, Fineberg HV, editors. Washington (DC): National Academies Press (US); 2024 Jul 9. 39110819

[21] Zigmond AS, Snaith RP. The hospital anxiety and depression scale. Acta Psychiatr Scand 1983 Jun;67(6):361–70. 10.1111/j.1600-0447.1983.tb09716.x 6880820

[22] Cohen S, Kamarck T, Mermelstein R. A global measure of perceived stress. J Health Soc Behav 1983 Dec;24(4):385–96. 10.2307/2136404 6668417

[23] Dambi JM, Corten L, Chiwaridzo M, Jack H, Mlambo T, Jelsma J. A systematic review of the psychometric properties of the cross-cultural translations and adaptations of the Multidimensional Perceived Social Support Scale (MSPSS). Health Qual Life Outcomes 2018 May;16(1):80. 10.1186/s12955-018-0912-0 29716589 PMC5930820

[24] Knol MJ, VanderWeele TJ. Recommendations for presenting analyses of effect modification and interaction. Int J Epidemiol 2012 Apr;41(2):514–20. 10.1093/ije/dyr218 22253321 PMC3324457

[25] Antonelli M, Pujol JC, Spector TD, Ourselin S, Steves CJ. Risk of long COVID associated with delta versus omicron variants of SARS-CoV-2. Lancet 2022 Jun;399(10343):2263–4. 10.1016/S0140-6736(22)00941-2 35717982 PMC9212672

[26] Bjorvatn B, Merikanto I, Reis C, Korman M, Bjelajac AK, Holzinger B et al. Shift workers are at increased risk of severe COVID-19 compared with day workers: results from the international COVID sleep study (ICOSS) of 7141 workers. Chronobiol Int 2023 Feb;40(2):114–22. 10.1080/07420528.2022.2148182 36412198

[27] Loef B, van der Beek AJ, Hulsegge G, van Baarle D, Proper KI. The mediating role of sleep, physical activity, and diet in the association between shift work and respiratory infections. Scand J Work Environ Health 2020 Sep;46(5):516–24. 10.5271/sjweh.3896 32255192 PMC7737798

[28] Galan R, Castaño-Vinyals G, Rubio-Garcia E, Lopez-Aladid R, Espinosa A, Papantoniou K et al. The Effect of Night Shift Work on the Gut Microbiome Diversity: the EXPONIT Study. J Biol Rhythms 2026 Jun;41(3):344–55. 10.1177/07487304251408152 41582394

[29] Papantoniou K, Konrad P, Haghayegh S, Strohmaier S, Eliassen AH, Schernhammer E. Rotating Night Shift Work, Sleep, and Thyroid Cancer Risk in the Nurses’ Health Study 2. Cancers (Basel) 2023;15(23):5673. 10.3390/cancers15235673 38067376 PMC10705158

[30] Tran DT, Strohmaier S, Żebrowska M, Huang T, Papantoniou K, Redline S et al. Night shift work and its interaction with sleep duration and chronotype, and risk of cardiovascular-kidney-metabolic diseases in the UK Biobank. Eur J Prev Cardiol 2025 Dec;(Dec):zwaf774. 10.1093/eurjpc/zwaf774 41411331

[31] Wright KP Jr, Bogan RK, Wyatt JK. Shift work and the assessment and management of shift work disorder (SWD). Sleep Med Rev 2013 Feb;17(1):41–54. 10.1016/j.smrv.2012.02.002 22560640

[32] Rizza S, Coppeta L, Grelli S, Ferrazza G, Chiocchi M, Vanni G et al. High body mass index and night shift work are associated with COVID-19 in health care workers. J Endocrinol Invest 2021 May;44(5):1097–101. 10.1007/s40618-020-01397-0 32852704 PMC7450678

[33] Nyberg supplementary table S, Frank P, Ahmadi-Abhari S, Pentti J, Vahtera J, Ervasti J et al. Adult obesity and risk of severe infections: a multicohort study with global burden estimates. Lancet 2026 Mar;407(10532):951–62. 10.1016/S0140-6736(25)02474-2. PMC12979006 41679324

[34] Afshin A, Forouzanfar MH, Reitsma MB, Sur P, Estep K, Lee A et al.; GBD 2015 Obesity Collaborators. Health Effects of Overweight and Obesity in 195 Countries over 25 Years. N Engl J Med 2017 Jul;377(1):13–27. 10.1056/NEJMoa1614362 28604169 PMC5477817

[35] Honce R, Schultz-Cherry S. Impact of Obesity on Influenza A Virus Pathogenesis, Immune Response, and Evolution. Front Immunol 2019 May;10:1071. 10.3389/fimmu.2019.01071 31134099 PMC6523028

[36] Kass DA, Duggal P, Cingolani O. Obesity could shift severe COVID-19 disease to younger ages. Lancet 2020 May;395(10236):1544–5. 10.1016/S0140-6736(20)31024-2 32380044 PMC7196905

[37] Zhou J, Li X, Zhang T, Liu Z, Li P, Yu N et al. Pre-existing sleep disturbances and risk of COVID-19: a meta-analysis. EClinicalMedicine 2024 Jul;74:102719. 10.1016/j.eclinm.2024.102719 39070174 PMC11276919

[38] Li P, Zheng X, Ulsa MC, Yang HW, Scheer FA, Rutter MK et al. Poor sleep behavior burden and risk of COVID-19 mortality and hospitalization. Sleep 2021 Aug;44(8):zsab138. 10.1093/sleep/zsab138 34142713 PMC8361340

[39] Peng L, Jing J, Ma J, He S, Gao X, Wang T. Insomnia and sleep duration on COVID-19 susceptibility and hospitalization: A Mendelian randomization study. Front Public Health 2022 Sep;10:995664. 10.3389/fpubh.2022.995664 36249224 PMC9561394

[40] Chen SJ, Morin CM, Ivers H, Wing YK, Partinen M, Merikanto I et al. The association of insomnia with long COVID: an international collaborative study (ICOSS-II). Sleep Med 2023 Dec;112:216–22. 10.1016/j.sleep.2023.09.034 37922783

[41] Vargas I, Muench A, Grandner MA, Irwin MR, Perlis ML. Insomnia symptoms predict longer COVID-19 symptom duration. Sleep Med 2023 Jan;101:365–72. 10.1016/j.sleep.2022.11.019 36493657 PMC9682867

[42] Livieratos A, Lockley SW, Tsiodras S. Post infectious fatigue and circadian rhythm disruption in long-COVID and other infections: a need for further research. EClinicalMedicine 2025 Jan;80:103073. 10.1016/j.eclinm.2025.103073 39896874 PMC11787434

[43] Komaroff AL, Lipkin WI. ME/CFS and Long COVID share similar symptoms and biological abnormalities: road map to the literature. Front Med (Lausanne) 2023 Jun;10:1187163. 10.3389/fmed.2023.1187163 37342500 PMC10278546

[44] Sengupta S, Brooks TG, Grant GR, FitzGerald GA. Accounting for Time: Circadian Rhythms in the Time of COVID-19. J Biol Rhythms 2021 Feb;36(1):4–8. 10.1177/0748730420953335 32875944 PMC8685580

[45] Erber AC, Wagner A, Karachaliou M, Jeleff M, Kalafatis P, Kogevinas M et al. The Association of Time of Day of ChAdOx1 nCoV-19 Vaccine Administration With SARS-CoV-2 Anti-Spike IgG Antibody Levels: An Exploratory Observational Study. J Biol Rhythms 2023 Feb;38(1):98–108. 10.1177/07487304221132355 36367167 PMC9659693

[46] Vink K, Kusters J, Wallinga J. Chrono-optimizing vaccine administration: a systematic review and meta-analysis. Front Public Health 2025 Apr;13:1516523. 10.3389/fpubh.2025.1516523 40260163 PMC12009823

[47] Wyse CA, Rudderham LM, Nordon EA, Ince LM, Coogan AN, Lopez LM. Circadian Variation in the Response to Vaccination: A Systematic Review and Evidence Appraisal. J Biol Rhythms 2024 Jun;39(3):219–36. 10.1177/07487304241232447 38459699 PMC11141079

[48] Spiegel K, Rey AE, Cheylus A, Ayling K, Benedict C, Lange T et al. A meta-analysis of the associations between insufficient sleep duration and antibody response to vaccination. Curr Biol 2023 Mar;33(5):998–1005.e2. 10.1016/j.cub.2023.02.017 36917932

[49] Scheiermann C, Kunisaki Y, Frenette PS. Circadian control of the immune system. Nat Rev Immunol 2013 Mar;13(3):190–8. 10.1038/nri3386 23391992 PMC4090048

[50] Cuesta M, Boudreau P, Dubeau-Laramée G, Cermakian N, Boivin DB. Simulated Night Shift Disrupts Circadian Rhythms of Immune Functions in Humans. J Immunol 2016 Mar;196(6):2466–75. 10.4049/jimmunol.1502422 26873990

[51] Harding BN, Aguilar R, Espinosa A, Castaño-Vinyals G, Papantoniou K, Navarrete JM et al. Disruption of cellular immune response among male rotating night shift workers in Spain- The HORMONIT study. Front Immunol 2022 Sep;13:776917. 10.3389/fimmu.2022.776917 36119067 PMC9478612

[52] Liu PY, Irwin MR, Krueger JM, Gaddameedhi S, Van Dongen HP. Night shift schedule alters endogenous regulation of circulating cytokines. Neurobiol Sleep Circadian Rhythms 2021 Mar;10:100063. 10.1016/j.nbscr.2021.100063 33748539 PMC7970107

[53] Papantoniou K, Pozo OJ, Espinosa A, Marcos J, Castaño-Vinyals G, Basagaña X et al. Circadian variation of melatonin, light exposure, and diurnal preference in day and night shift workers of both sexes. Cancer Epidemiol Biomarkers Prev 2014 Jul;23(7):1176–86. 10.1158/1055-9965.EPI-13-1271 24812038

[54] Lempesis IG, Georgakopoulou VE, Reiter RJ, Spandidos DA. A mid‑pandemic night’s dream: Melatonin, from harbinger of anti‑inflammation to mitochondrial savior in acute and long COVID‑19 (Review) [Review]. Int J Mol Med 2024 Mar;53(3):28. 10.3892/ijmm.2024.5352 38299237 PMC10852014

