# Supplementary material for: Night work, sleep disruption and long-COVID risk: a population-based cohort study
Source: Scand J Work Environ Health. 2026 Oct 26;52(6):689–701. doi: 10.5271/sjweh.4318 (PMC13734709; doi:10.5271/sjweh.4318)
Supplement: Supplementary file 1 [file SJWEH-52-6-689-s001.pdf]

Night work, sleep disruption and long-COVID risk: a population-based cohort study[1]

by Kyriaki Papantoniou, [2] Ana Espinosa, Marianna Karachaliou, Camille Lassale, Gemma Castaño-Vinyals, Barbara N Harding, Sara De Matteis, Kurt Straif, Eva Alonso Nogues, Carlota Dobaño, Gemma Moncunill, Natalia Blay, Susana Iraola-Guzmán, Rafael de Cid, PhD, Judith Garcia-Aymerich, Manolis Kogevinas

1. Supplementary materials
2. Correspondence to: Kyriaki Papantoniou, MD, MPH, PhD, Associate Professor, Medical University of Vienna, Center for Public Health, Department of Epidemiology, Spitalgasse 88, 1090, Vienna, Austria. [E-mail: kyriaki.papantoniou@meduniwien.ac.at]

Supplementary Methods – full list of long-COVID symptoms

Long-COVID definition: Among participants with a new SARS-CoV-2 infection, long-COVID was defined following NASEM [20] definition as (i) a self-report of at least one of the following symptoms or sequelae for a duration of at least 3 months which could not be explained by an alternative diagnosis based on responses in the 2023 questionnaire: fever or low-grade fever, loss of appetite, dry cough, dyspnoea/shortness of breath, persistent chest pain/tightness, throat pain, other respiratory problems or sequalae, tiredness/unusual fatigue, confusion/loss of speech/loss of movement, chills/vertigo/dizziness, loss of taste or smell, headache, other cognitive or neurological problems or sequelae, conjunctivitis, other problems with dry eyes/mouth, diarrhea, nausea/vomiting, skin rashes, other dermatological problems or sequelae, muscle/joint pain, other muscular problems or sequelae, hematological sequalae, endocrine sequalae, psychological or psychiatric sequelae, cardiovascular sequelae, menstrual problems, or renal problems or sequelae or (ii) identification of a COVID-19 sequelae (ICD-10 B94.8) or long-COVID diagnosis (U09.9) from EHR during the study period (2021-2023).

Supplementary Table S1. Most common occupations (classified according to ISCO-08) among night workers and comparison to day workers

ISCO-08	Job Title-08	Day workers	Night workers
5412	Police officers	6 (8.6)	8 (21.6)
2221	Nursing professionals	10 (14.3)	5 (13.5)
5321	Health care assistants	4 (5.7)	5 (13.5)
8211	Mechanical machinery assemblers	3 (4.3)	4 (10.8)
2212	Specialist medical practitioners	15 (21.4)	3 (8.1)
2423	Personnel and careers professionals	8 (11.4)	2 (5.4)
2635	Social work and counselling professionals	14 (20.0)	2 (5.4)
3114	Electronics engineering technicians	0 (0.0)	2 (5.4)
4222	Contact centre information clerks	8 (11.4)	2 (5.4)
5414	Security guards	1 (1.4)	2 (5.4)
9329	Manufacturing labourers not elsewhere classified	1 (1.4)	2 (5.4)

Supplementary Table S2. Sociodemographic characteristics (2021 survey) of the main study population (long-COVID analytic sample) by sleep duration and chronotype in the COVICAT cohort (N=1,899)

	**Sleep duration**			**Chronotype**		
	**Short (<6h)**	**Medium (6-9h)**	**Long (≥ 9h)**	**Early**	**Intermediate**	**Late**
Characteristic	**n=174 n(%)**	**n=1684 n(%)**	**n=41 n(%)**	**n=594 n(%)**	**n=673 n(%)**	**n=632 n(%)**
Age – mean (SD)	55.2 (6.1)	55.8 (6.9)	58.0 (9.1)	55.2 (6.6)	55.8 (7.1)	56.3 (7.0)
**Gender**						
Male	60 (34.5)	623 (37.0)	15 (36.6)	228 (38.4)	253 (37.6)	217 (34.3)
Female	114 (65.5)	1061 (63.0)	26 (63.4)	366 (61.6)	420 (62.4)	415 (65.7)
**Educational level**						
University	74 (42.5)	981 (58.3)	23 (56.1)	326 (54.9)	393 (58.4)	359 (56.8)
Secondary	81 (46.6)	586 (34.8)	14 (34.1)	216 (36.4)	237 (35.2)	228 (36.1)
Primary or less	19 (10.9)	117 (6.9)	4 (9.8)	52 (8.8)	43 (6.4)	45 (7.1)
**Smoking status**						
never smoker	71 (40.8)	730 (43.3)	19 (46.3)	260 (43.8)	294 (43.7)	266 (42.1)
ex-smoker	79 (45.4)	715 (42.5)	20 (48.8)	262 (44.1)	284 (42.2)	268 (42.4)
smoker	24 (13.8)	239 (14.2)	2 (4.9)	72 (12.1)	95 (14.1)	98 (15.5)
**Current alcohol consumption**						
No	45 (25.9)	366 (21.7)	16 (39.0)	142 (23.9)	150 (22.3)	135 (21.4)
Yes, occasionally	113 (64.9)	1104 (65.6)	23 (56.1)	391 (65.8)	429 (63.8)	420 (66.5)
Yes, daily	16 (9.2)	213 (12.7)	2 (4.9)	61 (10.3)	93 (13.8)	77 (12.2)
**Physical Activity, according to IPAQ scoring**						
Low	39 (22.4)	311 (18.5)	15 (36.6)	103 (17.3)	133 (19.8)	129 (20.4)
Moderate	74 (42.5)	809 (48.0)	21 (51.2)	270 (45.5)	314 (46.7)	320 (50.6)
High	61 (35.1)	564 (33.5)	5 (12.2)	221 (37.2)	226 (33.6)	183 (29.0)
**BMI**	26.9 (5.1)	26.2 (4.3)	28.0 (5.7)	26.2 (4.4)	26.2 (4.2)	26.6 (4.7)
**BMI categorical**						
<25	68 (39.1)	739 (43.9)	18 (43.9)	272 (45.8)	287 (42.6)	266 (42.1)
25-30	68 (39.1)	652 (38.7)	13 (31.7)	223 (37.5)	269 (40.0)	241 (38.1)
≥30	38 (21.8)	293 (17.4)	10 (24.4)	99 (16.7)	117 (17.4)	125 (19.8)

**COVID-19 severity among infected**						
Asymptomatic	33 (19.0)	291 (17.3)	6 (14.6)	113 (19.0)	115 (17.1)	102 (16.1)
Mild/Moderate	140 (80.5)	1387 (82.4)	35 (85.4)	480 (80.8)	557 (82.8)	525 (83.1)
Severe/Critical	1 (0.6)	6 (0.4)	0 (0.0)	1 (0.2)	1 (0.1)	5 (0.8)
**Vaccination doses**						
No vaccine	15 (8.8)	91 (5.5)	2 (5.0)	24 (4.1)	41 (6.2)	43 (6.9)
1 dose	0 (0.0)	20 (1.2)	1 (2.5)	6 (1.0)	7 (1.1)	8 (1.3)
2 doses	43 (25.1)	337 (20.3)	8 (20.0)	119 (20.3)	143 (21.5)	126 (20.3)
3-4 doses	113 (66.1)	1212 (73.0)	29 (72.5)	437 (74.6)	474 (71.3)	443 (71.5)
**Type of first vaccine**						
No vaccine	15 (8.6)	91 (5.4)	2 (4.9)	24 (4.0)	41 (6.1)	43 (6.8)
BioNTech	89 (51.1)	794 (47.1)	19 (46.3)	304 (51.2)	313 (46.5)	285 (45.1)
Moderna	26 (14.9)	193 (11.5)	3 (7.3)	65 (10.9)	86 (12.8)	71 (11.2)
Oxford	42 (24.1)	576 (34.2)	16 (39.0)	188 (31.6)	221 (32.8)	225 (35.6)
J&J / Sinovac	2 (1.1)	30 (1.8)	1 (2.4)	13 (2.2)	12 (1.8)	8 (1.3)
**Health state**						
Excellent	6 (3.4)	82 (4.9)	2 (4.9)	38 (6.4)	31 (4.6)	21 (3.3)
Very good	42 (24.1)	554 (32.9)	10 (24.4)	187 (31.5)	230 (34.2)	189 (29.9)
Good	80 (46.0)	841 (49.9)	18 (43.9)	291 (49.0)	331 (49.2)	317 (50.2)
Fair	40 (23.0)	194 (11.5)	8 (19.5)	72 (12.1)	73 (10.8)	97 (15.3)
Poor	6 (3.4)	13 (0.8)	3 (7.3)	6 (1.0)	8 (1.2)	8 (1.3)
Have a disease that required medical treatment for >6 months	69 (39.9)	544 (32.9)	24 (58.5)	204 (34.8)	214 (32.4)	219 (35.3)

Supplementary Table S3. Sociodemographic characteristics of the secondary study population (SARS-CoV-2 infection analytic outcome) by night work and chronic insomnia in the COVICAT cohort (N=2,941)

	**Night shift work**		**Chronic insomnia**	
	**No**	**Yes**	**No**	**Yes**
**Characteristic**	n=2801 n(%)	n=140 n(%)	n=2388 n(%)	n=553 n(%)
Age – mean (SD)	56.2 (7.0)	53.0 (6.3)	56.1 (7.1)	55.6 (6.3)
Gender				
Male	1081 (38.6)	91 (65.0)	1005 (42.1)	167 (30.2)
Female	1720 (61.4)	49 (35.0)	1383 (57.9)	386 (69.8)
Educational level				
University	1527 (54.5)	54 (38.6)	1318 (55.2)	263 (47.6)
Secondary	1053 (37.6)	74 (52.9)	894 (37.4)	233 (42.1)
Primary or less	221 (7.9)	12 (8.6)	176 (7.4)	57 (10.3)
Smoking status				
Never smoker	1197 (42.7)	59 (42.1)	1039 (43.5)	217 (39.2)
Ex-smoker	1162 (41.5)	59 (42.1)	983 (41.2)	238 (43.0)
Smoker	442 (15.8)	22 (15.7)	366 (15.3)	98 (17.7)
Current alcohol consumption				
No	636 (22.7)	32 (22.9)	503 (21.1)	165 (29.8)
Yes, ocasionally	1814 (64.8)	97 (69.3)	1588 (66.5)	323 (58.4)
Yes, daily	350 (12.5)	11 (7.9)	296 (12.4)	65 (11.8)
Physical Activity (IPAQ scoring)				
Low	510 (18.2)	22 (15.7)	411 (17.2)	121 (21.9)
Moderate	1332 (47.6)	59 (42.1)	1139 (47.7)	252 (45.6)
High	959 (34.2)	59 (42.1)	838 (35.1)	180 (32.5)
BMI – mean (SD)	26.3 (4.4)	26.8 (4.4)	26.3 (4.4)	26.2 (4.5)
BMI categorical				
<25	1232 (44.0)	53 (37.9)	1034 (43.3)	251 (45.4)
25-29.9	1087 (38.8)	57 (40.7)	937 (39.2)	207 (37.4)
≥30	482 (17.2)	30 (21.4)	417 (17.5)	95 (17.2)

COVID-19 severity among infected				
Asymptomatic	310 (17.1)	20 (22.5)	272 (17.7)	58 (15.9)
Mild/Moderate	1494 (82.5)	68 (76.4)	1257 (81.9)	305 (83.6)
Severe/Critical	6 (0.3)	1 (1.1)	5 (0.3)	2 (0.5)
Vaccination doses (pre-infection)				
No vaccine	116 (6.5)	5 (5.6)	95 (6.2)	26 (7.2)
1 dose	18 (1.0)	3 (3.4)	18 (1.2)	3 (0.8)
2 doses	367 (20.4)	21 (23.6)	306 (20.1)	82 (22.7)
3-4 doses	1294 (72.1)	60 (67.4)	1103 (72.5)	251 (69.3)
Type of first vaccine	*1006*	*51*	*866*	*191*
No vaccine	0 (0.0)	0 (0.0)	0 (0.0)	0 (0.0)
BioNTech	116 (4.1)	5 (3.6)	95 (4.0)	26 (4.7)
Moderna	1351 (48.2)	70 (50.0)	1147 (48.0)	274 (49.5)
Oxford	314 (11.2)	29 (20.7)	262 (11.0)	81 (14.6)
J&J / Sinovac	974 (34.8)	34 (24.3)	853 (35.7)	155 (28.0)
Health state	45 (1.6)	2 (1.4)	31 (1.3)	16 (2.9)
Excellent	0 (0.0)	0 (0.0)	0 (0.0)	0 (0.0)
Very good	1 (0.0)	0 (0.0)	0 (0.0)	1 (0.2)
Good	0 (0.0)	0 (0.0)	0 (0.0)	0 (0.0)
Fair	150 (5.4)	16 (11.4)	152 (6.4)	14 (2.5)
Poor	893 (31.9)	37 (26.4)	826 (34.6)	104 (18.8)

Supplementary Table S4. Association of night shift work, sleep disturbances and SARS-CoV-2 infection in the COVICAT cohort (2021-2023) (N=2,941)

	**No SARS-CoV-2 infection n (%)**	**SARS-CoV-2 Infection n (%)**	**RR (95% CI)[1]**	**RR (95% CI)[2]**
**NIGHT SHIFT WORK**	n=1,042	n=1,899		
Night shift work				
No	991 (95.1)	1810 (95.3)	ref.	ref.
Yes	51 (4.9)	89 (4.7)	1.02 (0.90-1.16)	1.02 (0.90-1.17)
Night shifts/month				
No night work	991 (95.1)	1810 (95.3)	ref.	ref.
<3 nights/month	16 (1.5)	19 (1.0)	0.85 (0.63-1.16)	0.84 (0.62-1.15)
≥3 nights/month	35 (3.4)	70 (3.7)	1.08 (0.94-1.24)	1.09 (0.95-1.25)
Night shift duration since March 2020				
No night work	991 (95.1)	1810 (95.3)	ref.	ref.
<3 months	14 (1.3)	27 (1.4)	1.06 (0.84 - 1.33)	1.05 (0.83-1.33)
≥3 months	37 (3.6)	62 (3.3)	1.01 (0.87-1.18)	1.01 (0.87-1.18)
**SLEEP**				
Sleep duration[3]				
<6h	108 (10.4)	174 (9.2)	0.97 (0.88-1.07)	0.97 (0.87-1.07)
6-7h	267 (25.6)	563 (29.6)	1.05 (0.99-1.12)	1.05 (0.99-1.12)
7-8h	433 (41.6)	783 (41.2)	ref.	ref.
8-9h	198 (19.0)	338 (17.8)	0.98 (0.91-1.06)	0.97 (0.90-1.05)
≥ 9h	36 (3.5)	41 (2.2)	0.84 (0.68-1.04)	0.83 (0.68-1.02)
Chronic insomnia				
No	854 (82.0)	1534 (80.8)	ref.	ref.
Yes	188 (18.0)	365 (19.2)	1.02 (0.95-1.09)	1.02 (0.95-1.09)
Sleep problems frequency				
No	670 (64.3)	1143 (60.2)	ref.	ref.
Yes, < 3 nights/week	184 (17.7)	391 (20.6)	1.06 (0.99-1.13)	1.06 (1.00-1.14)
Yes, ≥3nights/week	188 (18.0)	365 (19.2)	1.04 (0.97-1.11)	1.04 (0.97-1.11)
Sleep medication				
No	872 (83.7)	1541 (81.1)	ref.	ref.
Yes, natural treatment	80 (7.7)	189 (10.0)	1.07 (0.99-1.17)	1.07 (0.99-1.17)
Yes, medical treatment	90 (8.6)	169 (8.9)	1.02 (0.93-1.12)	1.03 (0.94-1.13)
Sleep timing (MSF)[3]				
Tertile 1 (Early)	394 (37.8)	594 (31.3)	ref.	ref.
Tertile 2 (Intermediate)	371 (35.6)	673 (35.4)	1.07 (1.00-1.14)	1.07 (1.00-1.14)
Tertile 3 (Late)	277 (26.6)	632 (33.3)	1.15 (1.08-1.23)	1.15 (1.08-1.23)

[1] Adjusted for age (continuous), sex and education (University/Secondary/Primary or less), [2] Adjusted for age (continuous), sex and education (University/Secondary/Primary or less), smoking (never/ex/current smoker), Physical activity according to IPAQ rules (low/moderate/high) and BMI (continuous), [3] Mid-sleep on free days (MSF) is calculated based on self-reported time of going to bed/waking up on a free day

Supplementary Table S5. Association of night shift work, sleep disturbances and long-COVID among SARS-CoV-2 infected participants in the COVICAT cohort (2021-2023) additionally adjusting for vaccine related factors, chronic diseases and SARS-CoV-2 infection severity (N=1,899)

	**RR (95% CI)[1]**	**RR (95% CI)[2]**	**RR (95% CI)[3]**
**NIGHT SHIFT WORK**			
No	ref.	ref.	ref.
Yes	1.82 (1.25-2.65)	1.79 (1.24-2.58)	1.89 (1.32-2.71)
Night shift work frequency			
No night work	ref.	ref.	ref.
<3 nights/month	1.25 (0.43-3.63)	1.12 (0.39-3.16)	1.17 (0.41-3.33)
≥3 nights/month	1.97 (1.34-2.89)	1.98 (1.37-2.87)	2.09 (1.45-3.02)
Night shift duration since March 2020			
No night work	ref.	ref.	ref.
<3 months	1.41 (0.61-3.28)	1.29 (0.59-2.82)	1.40 (0.63-3.12)
≥3 months	1.99 (1.34-2.95)	2.02 (1.37-2.97)	2.10 (1.44-3.07)
**SLEEP**			
Sleep duration			
<6h	1.33 (0.95-1.86)	1.31 (0.93-1.82)	1.35 (0.97-1.89)
6-7h	1.02 (0.78-1.32)	1.02 (0.79-1.32)	1.05 (0.81-1.35)
7-8h	ref.	ref.	ref.
8-9h	0.91 (0.66-1.26)	0.91 (0.66-1.27)	0.92 (0.67-1.28)
≥ 9h	1.60 (0.87-2.92)	1.49 (0.80-2.75)	1.58 (0.85-2.91)
Sleep problems frequency			
No	ref.	ref.	ref.
Yes, < 3 nights/week	1.49 (1.15-1.93)-	1.48 (1.14-1.92)-	1.47 (1.14-1.91)-
Yes, ≥3nights/week	1.64 (1.27-2.12)	1.53 (1.18-1.97)	1.59 (1.23-2.06)
Chronic insomnia			
No	ref.-	ref.-	ref.-
Yes	1.44 (1.14-1.83)-	1.34 (1.06-1.70)-	1.41 (1.11-1.78)-
Sleep medication			
No	ref.	ref.	ref.
Yes, natural treatment	1.54 (1.14-2.07)	1.50 (1.12-2.02)	1.53 (1.13-2.07)
Yes, medical treatment	1.24 (0.88-1.75)	1.09 (0.77-1.54)	1.21 (0.85-1.71)
Sleep timing/Chronotype (MSF)[3]			
Tertile 1 (Early)	ref.	ref.	ref.
Tertile 2 (Intermediate)	1.08 (0.82-1.41)	1.09 (0.83-1.44)	1.07 (0.81-1.40)
Tertile 3 (Late)	1.23 (0.94-1.60)	1.23 (0.94-1.60)	1.20 (0.92-1.57)

[1] Adjusted for age (continuous), sex and education (University/Secondary/Primary or less), smoking (never/ex/current smoker), Physical activity according to IPAQ rules (low/moderate/high), BMI (continuous), number of doses and type of vaccine , [2] Adjusted for age (continuous), sex and education (University/Secondary/Primary or less), smoking (never/ex/current smoker), Physical activity according to IPAQ rules (low/moderate/high), BMI (continuous) and chronic diseases (any), [3] Adjusted for age (continuous), sex and education (University/Secondary/Primary or less), smoking

(never/ex/current smoker), Physical activity according to IPAQ rules (low/moderate/high), BMI (continuous) and severity of COVID-19 (asymptomatic/mild, moderate, severe or critical),

Supplementary Table S6. Association of night shift work, sleep disturbances and long-COVID among SARS-CoV-2 infected participants in the COVICAT cohort (2021-2023) additionally adjusting for psychosocial factors (N=1,899)

NIGHT SHIFT WORK	RR (95% CI)[1]	RR (95% CI)[2]	RR (95% CI)[3]	RR (95% CI)[4]
No	ref.	ref.	ref.	ref.
Yes	1.78 (1.24-2.56)	1.85 (1.28-2.67)	1.80 (1.26-2.58)	1.73 (1.20-2.50)
Night shift work frequency				
No night work	ref.	ref.	ref.	ref.
<3 nights/month	1.08 (0.41-2.85)	1.05 (0.39-2.80)	1.14 (0.42-3.08)	1.16 (0.43-3.15)
≥3 nights/month	1.98 (1.37-2.88)	2.11 (1.45-3.06)	1.98 (1.37-2.86)	1.88 (1.28-2.75)
Night shift duration since March 2020				
No night work	ref.	ref.	ref.	ref.
<3 months	1.27 (0.57-2.83)	1.24 (0.55-2.81)	1.28 (0.58-2.85)	1.39 (0.64-3.03)
≥3 months	2.01 (1.38-2.94)	2.16 (1.48-3.14)	2.04 (1.41-2.96)	1.86 (1.26-2.76)
**SLEEP**				
Sleep duration				
<6h	1.13 (0.81-1.57)	1.04 (0.74-1.45)	1.07 (0.77-1.50)	1.19 (0.85-1.69)
6-7h	0.95 (0.73-1.23)	0.93 (0.71-1.21)	0.95 (0.73-1.24)	1.01 (0.78-1.31)
7-8h	ref.	ref.	ref.	ref.
8-9h	0.94 (0.68-1.30)	0.96 (0.70-1.33)	0.92 (0.67-1.27)	0.92 (0.66-1.27)
≥ 9h	1.55 (0.82-2.94)	1.60 (0.87-2.93)	1.41 (0.71-2.80)	1.49 (0.78-2.84)
Sleep problems frequency				
No	ref.	ref.	ref.	ref.
Yes, < 3 nights/week	1.32 (1.01-1.72)	1.27 (0.97-1.66)	1.30 (0.99-1.70)	1.42 (1.09-1.85)
Yes, ≥3nights/week	1.27 (0.97-1.66)	1.20 (0.90-1.59)	1.26 (0.96-1.67)	1.43 (1.09-1.89)
Chronic insomnia				
No	ref.	ref.	ref.	ref.
Yes	1.14 (0.89-1.46)	1.09 (0.84-1.40)	1.13 (0.88-1.46)	1.27 (0.98-1.63)
Sleep medication				
No	ref.	ref.	ref.	ref.
Yes, natural treatment	1.40 (1.04-1.88)	1.40 (1.04-1.89)	1.47 (1.10-1.98)	1.49 (1.11-2.01)
Yes, medical treatment	0.97 (0.68-1.39)	0.93 (0.65-1.33)	0.98 (0.68-1.40)	1.12 (0.79-1.59)
Sleep timing/Chronotype (MSF)[3]				
Tertile 1 (Early)	ref.	ref.	ref.	ref.
Tertile 2 (Intermediate)	1.10 (0.84-1.45)	1.10 (0.84-1.44)	1.11 (0.85-1.46)	1.12 (0.85-1.47)

Tertile 3 (Late)	1.23 (0.94-1.60)	1.21 (0.93-1.57)	1.22 (0.94-1.59)	1.23 (0.94-1.60)

[1] Adjusted for age (continuous), sex and education (University/Secondary/Primary or less), smoking (never/ex/current smoker), Physical activity according to IPAQ rules (low/moderate/high), BMI (continuous) **and perceived stress score (continuous)**

[2] Adjusted for age (continuous), sex and education (University/Secondary/Primary or less), smoking (never/ex/current smoker), Physical activity according to IPAQ rules (low/moderate/high), BMI (continuous) **and anxiety score from HADS (continuous)**

[3] Adjusted for age (continuous), sex and education (University/Secondary/Primary or less), smoking (never/ex/current smoker), Physical activity according to IPAQ rules (low/moderate/high), BMI (continuous) **and depression score from HADS (continuous)**

[4] Adjusted for age (continuous), sex and education (University/Secondary/Primary or less), smoking (never/ex/current smoker), Physical activity according to IPAQ rules (low/moderate/high), BMI (continuous) **and social support score (continuous)**

Supplementary Table S7. Association of night shift work, sleep disturbances and long-COVID among SARS-CoV-2 infected participants in the COVICAT cohort (2021-2023) restricting to participants with information on high risk job and additionally adjusting for high risk for COVID-19 job (N=1707)

	**No Long-COVID** n=1,454	**Long-COVID** n=253	**RR (95% CI)[1]**	**RR (95% CI)[2]**
**NIGHT SHIFT WORK**	**n (%)**	**n (%)**		
No	1396 (96.0)	237 (93.7)	ref.	ref.
Yes	58 (4.0)	16 (6.3)	1.69 (1.08-2.65)	1.67 (1.05-2.64)
Night shift work frequency				
No night work	1396 (96.0)	237 (93.7)	ref.	ref.
<3 nights/month	14 (1.0)	3 (1.2)	1.30 (0.45-3.76)	1.29 (0.45-3.76)
≥3 nights/month	44 (3.0)	13 (5.1)	1.82 (1.13-2.93)	1.79 (1.10-2.92)
Night shift duration since March 2020				
No night work	1396 (96.0)	237 (93.7)	ref.	ref.
<3 months	18 (1.2)	4 (1.6)	1.41 (0.56-3.59)	1.40 (0.54-3.60)
≥3 months	40 (2.8)	12 (4.7)	1.81 (1.11-2.95)	1.78 (1.08-2.93)
**SLEEP**				
Sleep duration				
<6h	118 (8.1)	32 (12.6)	1.43 (1.00-2.05)	1.43 (1.00-2.04)
6-7h	427 (29.4)	76 (30.0)	1.06 (0.80-1.39)	1.06 (0.80-1.39)
7-8h	615 (42.3)	97 (38.3)	ref.	ref.
8-9h	268 (18.4)	38 (15.0)	0.89 (0.63-1.25)	0.89 (0.63-1.26)
≥ 9h	26 (1.8)	10 (4.0)	1.85 (1.02-3.35)	1.86 (1.03-3.36)
Sleep problems frequency				
No	920 (63.3)	117 (46.2)	ref.	ref.
Yes, < 3 nights/week	282 (19.4)	68 (26.9)	1.57 (1.20-2.07)	1.57 (1.20-2.06)
Yes, ≥3nights/week	252 (17.3)	68 (26.9)	1.70 (1.29-2.24)	1.70 (1.29-2.23)
Chronic insomnia				
No	1202 (82.7)	185 (73.1)	ref.	ref.
Yes	252 (17.3)	68 (26.9)	1.46 (1.14-1.89)	1.46 (1.14-1.88)
Sleep medication				
No	1203 (82.7)	184 (72.7)	ref.	ref.
Yes, natural treatment	130 (8.9)	41 (16.2)	1.67 (1.22-2.27)	1.67 (1.23-2.27)
Yes, medical treatment	121 (8.3)	28 (11.1)	1.26 (0.88-1.82)	1.27 (0.88-1.83)
Sleep timing/Chronotype (MSF)[3]				
Tertile 1 (Early)	461 (31.7)	73 (28.9)	ref.	ref.
Tertile 2 (Intermediate)	528 (36.3)	88 (34.8)	1.03 (0.77-1.37)	1.03 (0.78-1.38)
Tertile 3 (Late)	465 (32.0)	92 (36.4)	1.15 (0.87-1.52)	1.15 (0.87-1.53)

Supplementary Table S8. Association of night work and sleep with long-COVID by chronotype, in the COVICAT cohort (2021-2023) (N=1899)

	Early Chronotype RR (95% CI)[1]	Intermediate Chronotype RR (95% CI)[1]	Late Chronotype RR (95% CI)[1]	Interaction p-value
NIGHT SHIFT WORK	n=594	n=673	n=632	
No	ref.	ref.	ref.	0.292
Yes	2.69 (1.40-5.17)	1.11 (0.44-2.76)	1.92 (1.16-3.17)	
SLEEP				
Sleep duration				
<6h	1.35 (0.75-2.41)	1.38 (0.75-2.55)	1.34 (0.76-2.35)	0.821
6-7h	0.91 (0.54-1.51)	1.11 (0.73-1.71)	1.11 (0.73-1.68)	
7-8h	ref.	ref.	ref.	
8-9h	0.77 (0.40-1.46)	0.82 (0.48-1.42)	1.21 (0.73-2.00)	
≥ 9h	0.61 (0.08-4.50)	0.75 (0.10-5.57)	2.45 (1.29-4.65)	
Sleep problems (>3 months)				
No	ref.	ref.	ref.	0.545
Yes	1.72 (1.12-2.64)	1.37 (0.90-2.08)	1.26 (0.86-1.84)	
Sleep problems frequency				
No, I don't have sleep problems	ref.	ref.	ref.	0.449
Yes, < 3 nights/week	1.82 (1.08-3.08)	1.72 (1.13-2.61)	1.13 (0.73-1.74)	
Yes, ≥3nights/week	2.06 (1.28-3.30)	1.66 (1.05-2.63)	1.31 (0.87-1.96)	
Sleep medication				
No	ref.	ref.	ref.	0.798
Yes, natural treatment	1.58 (0.86-2.91)	1.68 (1.02-2.74)	1.39 (0.87-2.21)	
Yes, medical treatment	1.42 (0.74-2.73)	0.87 (0.43-1.78)	1.34 (0.82-2.17)	

[1]Adjusted for age (continuous), sex, education (University/Secondary/Primary or less), smoking (never/ex/current smoker), Physical activity according to IPAQ rules (low/moderate/high) and BMI (continuous)

Supplementary Table S9. Association of sleep disturbances and long-COVID in the COVICAT cohort (2021-2023) excluding night shift workers (N= 1814)

	No long-COVID n=1,490	Long-COVID n=324	RR (95% CI)[1]	RR (95% CI)[2]
**SLEEP**				
**Sleep duration**				
<6h	129 (8.3)	32 (12.3)	1.38 (0.96-1.96)	1.30 (0.91-1.86)
6-7h	449 (29.0)	74 (28.4)	1.01 (0.77-1.33)	0.96 (0.73-1.26)
7-8h	657 (42.4)	104 (39.8)	ref.	ref.
8-9h	285 (18.4)	42 (16.1)	0.92 (0.66-1.29)	0.91 (0.65-1.27)
≥ 9h	29 (1.9)	9 (3.4)	1.74 (0.94-3.22)	1.53 (0.82-2.85)
**Chronic insomnia**				
No	1272 (82.1)	191 (73.2)	ref.	ref.
Yes	277 (17.9)	70 (26.8)	1.43 (1.12-1.84)	1.40 (1.09-1.79)
**Sleep problems frequency**				
No	967 (62.4)	125 (47.9)	ref.	ref.
Yes, < 3 nights/week	305 (19.7)	66 (25.3)	1.46 (1.11-1.92)	1.44 (1.10-1.89)
Yes, ≥3nights/week	277 (17.9)	70 (26.8)	1.61 (1.23-2.12)	1.57 (1.20-2.05)
**Sleep medication**				
No	1273 (82.2)	195 (74.7)	ref.	ref.
Yes, natural treatment	143 (9.2)	36 (13.8)	1.39 (1.00-1.93)	1.39 (1.00-1.92)
Yes, medical treatment	133 (8.6)	30 (11.5)	1.33 (0.94-1.88)	1.26 (0.88-1.80)
**Sleep timing (MSF)[3]**				
Tertile 1 (Early)	495 (32.0)	72 (27.6)	ref.	ref.
Tertile 2 (Intermediate)	553 (35.7)	93 (35.6)	1.16 (0.87-1.54)	1.14 (0.86-1.51)
Tertile 3 (Late)	501 (32.3)	96 (36.8)	1.28 (0.96-1.70)	1.24 (0.94-1.64)

[1] Adjusted for age (continuous), sex and education (University/Secondary/Primary or less)

[2] Adjusted for age (continuous), sex and education (University/Secondary/Primary or less), smoking (never/ex/current smoker), Physical activity according to IPAQ rules (low/moderate/high) and BMI (continuous)[3] Mid-sleep on free days (MSF) is calculated based on self-reported time of going to bed/waking up on a free day

Supplementary Table S10. Association of night work, sleep disturbances and long-COVID in the COVICAT cohort (2021-2023) restricting to participants who were employed in 2021 (N=1701)

	**No Long-COVID** n=1,456	**Long-COVID** n=245	**RR (95% CI)[1]**	**RR (95% CI)[2]**
**NIGHT SHIFT WORK**				
No	1397 (95.9)	229 (93.5)	ref.	ref.
Yes	59 (4.1)	16 (6.5)	1.67 (1.06-2.63)	1.68 (1.08-2.59)
Night shift work frequency				
No night work	1397 (95.9)	229 (93.5)	ref.	ref.
<3 nights/month	15 (1.0)	3 (1.2)	1.31 (0.47-3.65)	1.32 (0.46-3.77)
≥3 nights/month	44 (3.0)	13 (5.3)	1.78 (1.08-2.92)	1.79 (1.13-2.84)
Night shift duration since March 2020				
No night work	1397 (95.9)	229 (93.5)	ref.	ref.
<3 months	16 (1.1)	3 (1.2)	1.28 (0.44-3.72)	1.20 (0.42-3.46)
≥3 months	43 (3.0)	13 (5.3)	1.79 (1.10-2.91)	1.84 (1.17-2.90)
**SLEEP**				
Sleep duration				
<6h	122 (8.4)	28 (11.4)	1.34 (0.92-1.97)	1.26 (0.86-1.84)
6-7h	427 (29.3)	73 (29.8)	1.06 (0.81-1.40)	1.03 (0.78-1.35)
7-8h	615 (42.2)	97 (39.6)	ref.	ref.
8-9h	266 (18.3)	39 (15.9)	0.93 (0.66-1.32)	0.93 (0.66-1.30)
≥ 9h	26 (1.8)	8 (3.3)	1.76 (0.91-3.40)	1.59 (0.83-3.07)
Sleep problems frequency				
No	920 (63.2)	121 (49.4)	ref.	ref.
Yes, < 3 nights/week	283 (19.4)	64 (26.1)	1.49 (1.13-1.96)	1.48 (1.13-1.95)
Yes, ≥3nights/week	253 (17.4)	60 (24.5)	1.53 (1.15-2.05)	1.51 (1.13-2.00)
Chronic insomnia				
No	1203 (82.6)	185 (75.5)	ref.	ref.
Yes	253 (17.4)	60 (24.5)	1.36 (1.04-1.77)	1.33 (1.02-1.74)
Sleep medication				
No	1202 (82.6)	180 (73.5)	ref.	ref.
Yes, natural treatment	133 (9.1)	39 (15.9)	1.59 (1.16-2.19)	1.60 (1.17-2.19)
Yes, medical treatment	121 (8.3)	26 (10.6)	1.28 (0.88-1.86)	1.20 (0.82-1.74)
Sleep timing/Chronotype (MSF)[3]				
Tertile 1 (Early)	461 (31.7)	69 (28.2)	ref.	ref.
Tertile 2 (Intermediate)	524 (36.0)	87 (35.5)	1.11 (0.83-1.49)	1.10 (0.82-1.47)
Tertile 3 (Late)	471 (32.3)	89 (36.3)	1.22 (0.91-1.64)	1.16 (0.87-1.55)

[1] Adjusted for age (continuous), sex and education (university/secondary/primary or less)

[2] Adjusted for age (continuous), sex and education (university/secondary/primary or less), smoking (never/ex/current smoker), physical activity according to IPAQ rules (low/moderate/high) and body mass index (continuous)

[3] Mid-sleep on free days (MSF) is calculated based on self-reported time of going to bed/waking up on a free day corrected for overlseep

Supplementary Table S11. Association of night work, sleep disturbances and long-COVID in the COVICAT cohort (2021-2023) restricting to mild/moderate severity infections (N=1596)

	No long-COVID n=1,335 n (%)	Long-COVID n=261 n (%)	RR (95% CI)[1]	RR (95% CI)[2]
**NIGHT SHIFT WORK**				
No	1264 (96.4)	230 (91.6)	ref.	ref.
Yes	47 (3.6)	21 (8.4)	2.19 (1.50-3.18)	2.18 (1.51-3.14)
**Night shift work frequency**				
No night work	1264 (96.4)	230 (91.6)	ref.	ref.
<3 nights/month	13 (1.0)	3 (1.2)	1.34 (0.49-3.69)	1.33 (0.47-3.72)
≥3 nights/month	34 (2.6)	18 (7.2)	2.45 (1.66-3.63)	2.44 (1.68-3.54)
**Night shift duration since March 2020**				
No night work	1264 (96.4)	230 (91.6)	ref.	ref.
<3 months	17 (1.3)	5 (2.0)	1.68 (0.76-3.70)	1.59 (0.71-3.55)
≥3 months	30 (2.3)	16 (6.4)	2.41 (1.61-3.62)	2.45 (1.68-3.59)
**SLEEP**				
**Sleep duration**				
<6h	110 (8.4)	30 (12.0)	1.27 (0.88-1.82)	1.21 (0.84-1.74)
6-7h	385 (29.4)	74 (29.5)	1.01 (0.77-1.32)	0.97 (0.74-1.27)
7-8h	551 (42.0)	101 (40.2)	ref.	ref.
8-9h	238 (18.2)	38 (15.1)	0.87 (0.61-1.22)	0.84 (0.60-1.18)
≥ 9h	27 (2.1)	8 (3.2)	1.48 (0.76-2.88)	1.31 (0.66-2.59)
**Sleep problems (>3 months)**				
No	808 (61.6)	118 (47.0)	ref.	ref.
Yes, < 3 nights/week	264 (20.1)	67 (26.7)	1.50 (1.14-1.97)	1.48 (1.12-1.94)
Yes, ≥3nights/week	239 (18.2)	66 (26.3)	1.54 (1.17-2.02)	1.51 (1.15-1.98)
**Chronic insomnia**				
No	1072 (81.8)	185 (73.7)	ref.	ref.
Yes	239 (18.2)	66 (26.3)	1.35 (1.05-1.74)	1.32 (1.03-1.70)
**Sleep medication**				
No	1075 (82.0)	187 (74.5)	ref.	ref.
Yes, natural treatment	122 (9.3)	38 (15.1)	1.46 (1.06-2.01)	1.46 (1.07-2.01)
Yes, medical treatment	114 (8.7)	26 (10.4)	1.19 (0.82-1.73)	1.12 (0.77-1.64)
**Sleep timing/Chronotype (MSF)[3]**				
Tertile 1 (Early)	410 (31.3)	70 (27.9)	ref.	ref.
Tertile 2 (Intermediate)	473 (36.1)	84 (33.5)	1.05 (0.79-1.41)	1.02 (0.76-1.36)
Tertile 3 (Late)	428 (32.6)	97 (38.6)	1.27 (0.96-1.69)	1.21 (0.92-1.60)

[1] Adjusted for age (continuous), sex and education (University/Secondary/Primary or less)

[2] Adjusted for age (continuous), sex and education (University/Secondary/Primary or less), smoking (never/ex/current smoker), Physical activity according to IPAQ rules (low/moderate/high) and BMI (continuous)[3] Mid-sleep on free days (MSF) is calculated based on self-reported time of going to bed/waking up on a free day

Supplementary Table S12. Association of night work, sleep disturbances and long-COVID in the COVICAT cohort (2021-2023) restricting to those with >2 symptoms (N=1736)

	**No Long-COVID** n=1,619	**Long-COVID** n=117	**RR (95% CI)[1]**	**RR (95% CI)[2]**
**NIGHT SHIFT WORK**	**n (%)**	**n (%)**		
No	1549 (95.9)	108 (92.3)	ref.	ref.
Yes	67 (4.1)	9 (7.7)	1.88 (0.96-3.68)	1.88 (1.01-3.51)
Night shift work frequency				
No night work	1549 (95.9)	108 (92.3)	ref.	ref.
<3 nights/month	16 (1.0)	2 (1.7)	1.80 (0.47-6.86)	1.75 (0.47-6.50)
≥3 nights/month	51 (3.2)	7 (6.0)	1.91 (0.91-4.00)	1.93 (0.99-3.76)
Night shift duration since March 2020				
No night work	1549 (95.9)	108 (92.3)	ref.	ref.
<3 months	22 (1.4)	2 (1.7)	1.47 (0.38-5.64)	1.38 (0.37-5.16)
≥3 months	45 (2.8)	7 (6.0)	2.05 (0.98-4.28)	2.11 (1.09-4.08)
**SLEEP**				
Sleep duration				
<6h	138 (8.5)	18 (15.4)	1.78 (1.06-3.00)	1.69 (1.00-2.83)
6-7h	477 (29.5)	35 (29.9)	1.11 (0.72-1.70)	1.03 (0.67-1.58)
7-8h	676 (41.8)	42 (35.9)	ref.	ref.
8-9h	294 (18.2)	18 (15.4)	0.99 (0.58-1.70)	0.96 (0.56-1.63)
≥ 9h	31 (1.9)	4 (3.4)	2.05 (0.73-5.71)	1.74 (0.62-4.90)
Sleep problems frequency				
No	1010 (62.5)	39 (33.3)	ref.	ref.
Yes, < 3 nights/week	318 (19.7)	35 (29.9)	2.54 (1.63-3.95)	2.51 (1.62-3.89)
Yes, ≥3nights/week	288 (17.8)	43 (36.8)	3.12 (2.03-4.78)	3.03 (1.99-4.62)
Chronic insomnia				
No	1328 (82.2)	74 (63.2)	ref.	ref.
Yes	288 (17.8)	43 (36.8)	2.20 (1.53-3.17)	2.15 (1.50-3.08)
Sleep medication				
No	1332 (82.4)	84 (71.8)	ref.	ref.
Yes, natural treatment	146 (9.0)	16 (13.7)	1.54 (0.90-2.63)	1.54 (0.91-2.61)
Yes, medical treatment	138 (8.5)	17 (14.5)	1.80 (1.11-2.92)	1.62 (0.98-2.67)
Sleep timing/Chronotype (MSF)[3]				
Tertile 1 (Early)	515 (31.9)	37 (31.6)	ref.	ref.
Tertile 2 (Intermediate)	576 (35.6)	33 (28.2)	0.85 (0.54-1.34)	0.82 (0.52-1.28)
Tertile 3 (Late)	525 (32.5)	47 (40.2)	1.29 (0.86-1.95)	1.19 (0.79-1.79)

[1] Adjusted for age (continuous), sex and education (University/Secondary/Primary or less)

[2] Adjusted for age (continuous), sex and education (University/Secondary/Primary or less), smoking (never/ex/current smoker), Physical activity according to IPAQ rules (low/moderate/high) and BMI (continuous)[3] Mid-sleep on free days (MSF) is calculated based on self-reported time of going to bed/waking up on a free day

Supplementary Table S13. Characteristics of included (N=1899) and excluded (N=128) participants in the long-COVID analyses in the COVICAT cohort (2021-2023)

Characteristic	Non-responders n=128 n(%)	Responders n=1899 n(%)
Age	37 (28.9)	421 (22.2)
<50	82 (64.1)	1236 (65.1)
50-64	9 (7.0)	242 (12.7)
Gender		
Male	40 (31.2)	698 (36.8)
Female	88 (68.8)	1201 (63.2)
Educational level		
University	65 (50.8)	1078 (56.8)
Secondary	56 (43.8)	681 (35.9)
Primary or less	7 (5.5)	140 (7.4)
Smoking status		
Never smoker	33 (33.7)	820 (43.2)
Ex-smoker	46 (46.9)	814 (42.9)
Smoker	19 (19.4)	265 (14.0)
Current alcohol consumption		
No	20 (20.4)	427 (22.5)
Yes, occasionally	64 (65.3)	1240 (65.3)
Yes, daily	14 (14.3)	231 (12.2)
Physical Activity (IPAQ scoring)		
Low	13 (20.6)	365 (19.2)
Moderate	29 (46.0)	904 (47.6)
High	21 (33.3)	630 (33.2)
BMI – mean (SD)	26.5 (4.8)	26.3 (4.4)
BMI categorical		
<25	46 (40.4)	825 (43.4)
25-29.9	45 (39.5)	733 (38.6)
≥30	23 (20.2)	341 (18.0)
COVID-19 severity among infected		
Asymptomatic	23 (18.0)	330 (17.4)
Mild/Moderate	103 (80.5)	1562 (82.3)
Severe/Critical	2 (1.6)	7 (0.4)
Vaccination doses (pre-infection)		
No vaccine	8 (6.2)	108 (5.8)
1 dose	3 (2.3)	21 (1.1)
2 doses	31 (24.2)	388 (20.7)
3-4 doses	86 (67.2)	1354 (72.4)
Type of first vaccine		
No vaccine	8 (6.2)	108 (5.7)
BioNTech	70 (54.7)	902 (47.5)
Moderna	19 (14.8)	222 (11.7)
Oxford	28 (21.9)	634 (33.4)
J&J / Sinovac	3 (2.3)	33 (1.7)
Health state		
Excellent	2 (4.9)	90 (4.7)

Very good	9 (22.0)	606 (31.9)
Good	25 (61.0)	939 (49.4)
Fair	3 (7.3)	242 (12.7)
Poor	2 (4.9)	22 (1.2)
Disease that required medical treatment for >6 months	15 (36.6)	637 (34.1)
